# Indoor VOCs Exposure Disrupts Hemostatic Balance: Plasma Proteomics Identifies Complement and Coagulation Cascade Dysregulation as a Critical Signature

**DOI:** 10.1021/envhealth.5c00641

**Published:** 2026-03-19

**Authors:** Yurong Cui, Gan Miao, Duxing Li, Tian Qiu, Peijie Sun, Yuebin Lv, Xiaohan Sun, Yijiao Li, Rong Zhang, Lin Lu, Xiaoting Jin, Yuxin Zheng

**Affiliations:** † Department of Occupational Health and Environmental Health, School of Public Health, 12593 Qingdao University, Qingdao 266071, China; ‡ China CDC Key Laboratory of Environment and Population Health, Chinese Center for Disease Control and Prevention, National Institute of Environmental Health, Beijing 100021, China; § Department of Toxicology, School of Public Health, 12553 Hebei Medical University, Shijiazhuang 050017, China; ∥ ZaoZhuang Center for Disease Control and Prevention, Zaozhuang 277101, China

**Keywords:** volatile organic compounds, complement and coagulation cascades, plasma proteomics, hematological disturbance, risk score model

## Abstract

Volatile organic compounds (VOCs) are prevalent indoor pollutants, posing significant health risks. However, current toxicological data focus predominantly on individual compounds, leaving a critical gap in understanding how exposure to mixed VOCs alters the plasma proteome and impacts health risk prediction. Herein, we investigated VOCs-induced hematological disturbances using a whole-body inhalation exposure model with C57BL/6J mice (*n* = 3) exposed to decoration-derived VOCs for 8 weeks. By integrating plasma proteomics with machine learning, we demonstrated that VOCs exposure significantly disrupts hemostatic homeostasis, primarily by perturbing the complement and coagulation cascades. Network enrichment and correlation analyses further identified a network of suppressed pathways (e.g., platelet activation and focal adhesion) and activated pathways (e.g., necroptosis and IL-17 signaling pathway) that collectively drive this dysregulation. Critically, we identified downregulated von Willebrand factor (VWF) and cell division control protein 42 (CDC42) as a robust biomarker signature, as evidenced by correlation coefficients (*r*) of 0.91 and 0.90 with complement and coagulation cascades, respectively. Leveraging these biomarkers, we developed a highly accurate health risk scoring model. This study provides the first comprehensive molecular portrait of VOCs-induced hematological disturbances and presents a novel proteomics-driven framework for the early prediction and intervention of VOCs-related health effects.

## Graphical abstract

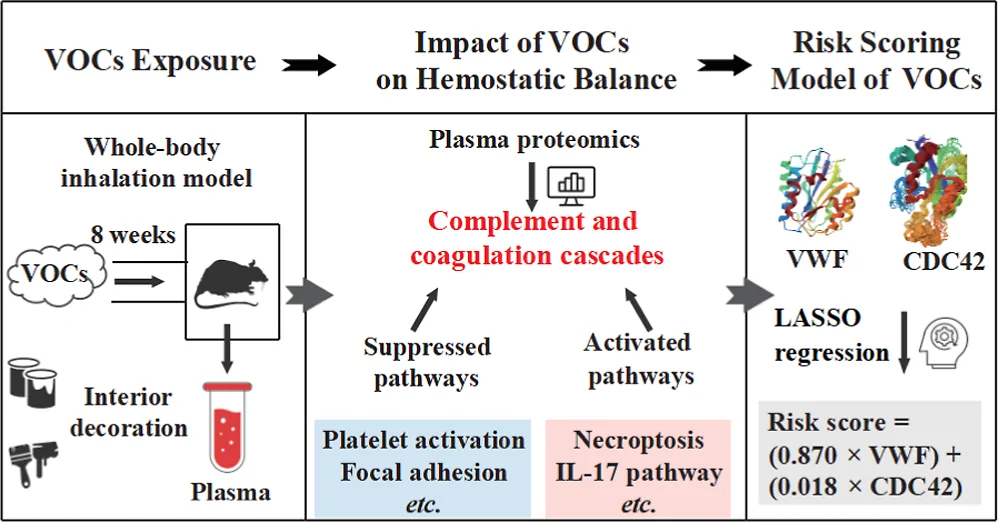

## 1 Introduction

Air pollution has become a critical and growing public health challenge worldwide.^1^ According to the Global Burden of Disease 2021 (GBD2021) study, exposure to indoor air pollution contributes to approximately 3.11 million deaths annually.^2^ Given that individuals spend approximately 80% of their time in indoor environments, indoor air constitutes a major route of exposure to airborne pollutants.^3^ Notably, elevated concentrations of indoor volatile organic compounds (VOCs) are often twice as high as outdoor levels due to inadequate ventilation and enhanced building airtightness, posing a significant health concern.^4,5^ For example, the average indoor concentrations of toluene and ethylbenzene are 11 μg/m^3^ and 5.6 μg/m^3^, respectively, compared to outdoor averages of 5.5 μg/m^3^ and 1.6 μg/m^3^.^6,7^ Multiple studies have demonstrated that building materials and furnishings serve as persistent sources of indoor VOCs emissions, contributing up to 90% of the total indoor VOCs levels.^8–10^ The widespread use of modern construction materials and indoor furnishings has further aggravated this problem, contributing to significantly higher indoor VOCs emissions. Consequently, the health implications of indoor VOCs pollution have attracted growing attention. Epidemiological research has revealed a strong correlation between VOCs exposure and the development of leukemia.^11^ For each 1 μg/m^3^ increase in the concentration of VOCs, the pooled relative risk for leukemia is 1.25.^12^ Multiple VOCs components have been linked to hematological abnormalities, such as the impairment of hematopoiesis.^13^ Moreover, chronic exposure to VOCs has been associated with a range of adverse health effects, particularly affecting the cardiovascular, hepatic, and respiratory systems.^14–16^ Despite these well-documented risks, a critical gap persists in identifying validated susceptibility and effect biomarkers for adverse outcomes from exposure to VOCs, highlighting an urgent need for further investigation.

Blood analysis offers a comprehensive assessment of individual exposure, health effects, and susceptibility by simultaneously detecting exogenous compounds, endogenous metabolites, and key biomarkers of inflammation and damage.^17,18^ Unlike urinary markers, blood biomarkers provide a superior representation of systemic responses by integrating cumulative exposures and reflecting real-time pathophysiological changes.^19^ However, establishing reliable plasma effect biomarkers for VOCs exposure in epidemiological studies is fraught with challenges, creating a significant research dilemma. On one hand, population-level investigations are substantially confounded by ubiquitous sources of VOCs, most notably smoking, which introduces considerable variability and bias, thereby hindering accurate risk characterization.^20,21^ On the other hand, conventional toxicological models, which inform the interpretation of these biomarkers, often fall short of reflecting real-world exposure scenarios. These models typically rely on individual VOCs or simplified artificial mixtures administered at acute, high doses—frequently exceeding occupational thresholds. Such approaches fail to recapitulate the complex, low-dose, chronic exposures characteristic of indoor environments, where potential synergistic interactions among numerous chemicals are overlooked.^22,23^ This disconnection between the limitations of observational epidemiology and the reduced translational relevance of experimental models creates a critical knowledge gap. Therefore, there is an urgent need to investigate the health risks of VOCs exposure using plasma biomarkers within the context of real-world exposure models.

Plasma proteins have been widely recognized as important biomarkers for reflecting physiological and pathological states in humans.^24^ Their dynamic expression levels provide critical clues for understanding the onset and progression of diseases. Plasma proteomics offers a powerful approach for uncovering protein biomarkers, thereby helping bridge the knowledge gap between protein expression and disease phenotypes.^25,26^ A systematic assessment of alterations in the plasma proteome in response to exogenous exposures provides a critical means for comprehensively understanding health risks induced by environmental stimuli.^27,28^ However, current research utilizing plasma proteomics to assess environmental pollutant exposure remains predominantly limited to differential protein expression analysis and pathway annotation, thus overlooking the potential value of plasma proteins as validated biomarkers. For example, while some studies have identified VOCs-associated protein expression changes, few have rigorously evaluated their diagnostic or prognostic utility as biomarkers.^29^ Furthermore, although certain studies have revealed associations between specific proteins and environmental exposure through plasma proteomic analyses, they often fail to integrate these findings into risk score models, thereby limiting the clinical application and practical significance of the biomarkers.^30–32^ Consequently, the field has yet to determine which protein combinations—or optimal plasma protein biosignatures—can accurately predict individual susceptibility to VOCs-induced health effects.

This study builds upon our previously established whole-body inhalation exposure mouse model for indoor decoration-derived VOCs.^15^ We employed plasma proteomics to screen for the key proteins and pathways. Subsequently, a machine learning approach (the least absolute shrinkage and selection operator, LASSO) was used to identify a core biomarker panel and construct a predictive risk score model. This model’s performance was then validated in an in *vivo* model. By integrating biomarker discovery with machine learning, this study provides a combinatorial protein signature to accurately forecast individual health risks from VOCs exposure. Our findings are expected to provide profound insights into the hematological disturbances induced by interior decoration-derived VOCs and to inform the development of targeted interventions for mitigating their adverse effects.

## 2 Materials and Methods

### 2.1 Animal Experiment and VOCs Exposure

6 weeks old male C57BL/6J mice (22.2 ± 1.1 g), purchased from Vital River Laboratory (Beijing, China), were housed in the standard animal center of Hebei Medical University under controlled conditions (23 °C, 12 h light–dark cycle). All mice were fed a standard pellet diet and were provided free access to water. After a 1 W acclimatization period, mice (*n* = 3 per group) were randomly allocated to either control (CON) or VOCs exposure groups.

Mice were exposed to filtered emissions from decorative paint volatiles (Shijiazhuang Paint Factory, Shijiazhuang, China) for 8 hours (h) daily over 8 W, as previously described.^15^ A custom-designed cage-based exposure system was utilized to simulate indoor-relevant VOCs conditions. The system consisted of an air blower that pumped indoor air through filter membranes into the mouse cages. Control animals received air filtered through both High-Efficiency Particulate Air (HEPA) and activated carbon filters (AST, China Environmental Technology Co., Ltd.) to remove particulate matter and VOCs, respectively. The VOCs group received HEPA-filtered air with particulate matter removed, but VOCs were retained. Exposure conditions were maintained at a constant temperature (25 ± 1 °C), relative humidity (50% ± 5%), and pressure differential (25 kPa) throughout the experimental period. Experimental procedures were approved by the Animal Care and Use Committee of Qingdao University (QDU-AEC-2024543) and Hebei Medical University (IACUC-Hebmu-20170116), carried out under the standards for ethical animal usage. All mice were euthanized via intraperitoneal injection of sodium pentobarbital following final exposure. Blood plasma was collected for a subsequent proteomic analysis.

### 2.2 Sample Preparation and Pretreatment

#### 2.2.1 Sample Preparation

Cell or tissue samples were suspended in 200 μL of lysis buffer (4% SDS, 100 mmol/L DTT, and 150 mmol/L *Tris*–HCl, pH 8.0) on ice. The cells or tissues were thoroughly lysed using a homogenizer under vigorous shaking, followed by boiling the samples for 5 minutes (min). To ensure complete protein denaturation and DNA fragmentation, the samples were subjected to ultrasonication and boiled again for 5 min. Insoluble debris was removed by centrifugation at 16,000 rpm for 15 min. The supernatant was collected, and the protein concentration was determined using a BCA Protein Assay Kit (Bio-Rad, USA).

#### 2.2.2 Protein Digestion

Briefly, the detergent (SDS), DTT, and other small-molecule impurities were thoroughly removed by multiple centrifugal exchanges using 200 μL of UA buffer (8 mol/L Urea, 150 mmol/L *Tris*–HCl, pH 8.0) in a 30 kDa ultrafiltration centrifugal unit (Microcon). Subsequently, 100 μL of UA buffer containing 0.05 mol/L iodoacetamide (IAA) was added and incubated in the dark for 20 min to block the reduced cysteine residues. The filter membrane was washed 3× with 100 μL of UA buffer and twice with 100 μL of 25 mmol/L NH_4_HCO_3_ buffer. Finally, the protein suspension was digested with 4 μg of trypsin (Promega) in 40 μL of 25 mmol/L NH_4_HCO_3_ at 37 °C overnight, and the resulting peptides were collected as a filtrate. Peptide concentration was determined by measuring OD_280_ by using a Nanodrop device.

#### 2.2.3 TMT Labeling of Peptides

TMT labeling of peptide samples was strictly performed according to the manufacturer’s (Thermo Fisher Scientific) instructions. A total of 100 μg of peptides was dissolved in 100 μL of 50 mmol/L TEAB (pH 8.5). The TMT reagent was dissolved in 41 μL of anhydrous acetonitrile and then added to the peptide solution. The mixture was incubated at room temperature for 1 h. The reaction was quenched by adding 8 μL of a 5% hydroxylamine solution and incubating for an additional 15 min. The multiplex-labeled samples were pooled together and lyophilized.

#### 2.2.4 High-pH Reverse Phase Fractionation

TMT-labeled peptide mixtures were separated by using a Waters XBridge BEH130 column (C18, 3.5 μm, 2.1 × 150 mm) on an Agilent 1290 high-performance liquid chromatography system at a flow rate of 0.3 mL/min. The mobile phase consisted of buffer A (10 mmol/L ammonium formate) and buffer B (10 mmol/L ammonium formate in 90% acetonitrile), both adjusted to pH 10 with ammonium hydroxide. A total of 30 fractions were collected from each peptide mixture and subsequently combined into 15 fractions by merging every other fraction in the reversed-phase elution profile. The fractions were dried and prepared for nanoliquid chromatography–tandem mass spectrometry (nLC–MS/MS) analysis.

### 2.3 Proteomic Analysis

#### 2.3.1 LC–MS Analysis

LC–MS/MS analysis was performed on a Q Exactive mass spectrometer coupled to an Easy nLC system (Thermo Fisher Scientific). Peptides from each fraction were loaded onto a C18 reversed-phase column (12 cm long, 75 μm ID, 3 μm particles) with buffer A (2% acetonitrile and 0.1% formic acid) and separated at a flow rate of 300 nL/min over a 90 min linear gradient of buffer B (90% acetonitrile and 0.1% formic acid). The gradient was programed as follows: 0–2 min, 2% to 5% B; 2–62 min, 5% to 20% B; 62–80 min, 20% to 35% B; 80–83 min, 35% to 90% B; 83–90 min, and 90% B held isocratically. Mass spectrometry data were acquired using a data-dependent top-15 method, dynamically selecting the most abundant precursor ions from the full scan (range: 300–1800 *m*/*z*) for fragmentation via higher-energy collisional dissociation (HCD). The automatic gain control (AGC) target was set based on predictive AGC (pAGC). The full MS AGC target was 1e^6^ with a maximum injection time of 50 ms, while the MS^2^ AGC target was 1e^5^ with a maximum injection time of 100 ms. The dynamic exclusion duration was set to 30 s. The survey scan resolution was set to 70,000 at *m*/*z* 200, and the HCD spectral resolution was 35,000. The normalized collision energy was 30. The peptide recognition mode was enabled during the instrument operation.

#### 2.3.2 Principal Component Analysis

The principal component analysis (PCA) method based on the R package “FactoMineR” and “factoextra” was employed to assess the extent of differences between the exposed and control groups, with statistical significance defined at *P* < 0.05. Intragroup reproducibility was evaluated based on sample expression distribution and intersample correlation.

### 2.4 Bioinformatics Analysis

#### 2.4.1 Differential Expression Analysis

Bioinformatic analyses were conducted using Proteome Discoverer 2.4 software (version 1.6.0.16), Perseus software (version 2.0), Microsoft Excel, RStudio (version 4.4), and relevant R packages. Differentially expressed proteins (DEPs) were identified using fold change (FC) thresholds of |log_2_FC| > 0.263 (equivalent to FC > 1.2 or FC < 1/1.2) and statistical significance (*P* < 0.05). Volcano plots were generated using the ggplot2 package to visualize DEPs, while hierarchical clustering analysis was performed using the pheatmap package to group samples based on protein expression patterns.^33^

#### 2.4.2 Functional Enrichment Analysis

Functional annotation of DEPs was performed by using the clusterProfiler package. Gene Ontology (GO) enrichment analysis was conducted across three categories: biological process (BP), molecular function (MF), and cellular component (CC). Subcellular localization was determined by CC analysis. Pathway enrichment analyses were performed using the Kyoto Encyclopedia of Genes and Genomes (KEGG) and Reactome databases (https://www.reactome.org). Statistical significance for all enrichment analyses was determined using Fisher’s exact test with false discovery rate (FDR) correction for multiple comparisons. Enrichment terms with FDR < 0.05 were considered statistically significant. Functional enrichment networks of KEGG pathways were visualized using the enrichplot package, depicting the remaining differentially regulated proteins following the exclusion of the complement and coagulation cascades, with pathways related to human diseases also being further excluded.

#### 2.4.3 Gene Set Enrichment Analysis

To evaluate pathway-level alterations under VOCs exposure, Gene Set Enrichment Analysis (GSEA) was performed as previously described.^34^ Genes were ranked based on differential expression, and enrichment scores were calculated using the clusterProfiler package. Statistical significance was assessed with FDR correction, where normalized enrichment scores (NES) with |NES| > 1 and FDR < 0.25 were considered significant. GSEA results were visualized using the enrichplot package.

#### 2.4.4 Gene Set Variation Analysis

Gene Set Variation Analysis (GSVA) was implemented as an unsupervised, nonparametric method to evaluate pathway activity variations across samples.^35^ Using the GSVA package, enrichment scores were calculated for each sample within predefined gene sets, generating a comprehensive enrichment matrix. The resulting matrix was visualized as a heatmap using the pheatmap package.

#### 2.4.5 Protein–Protein Interaction Network Analysis

Protein–protein interactions (PPI) were analyzed using the STRING database (https://www.string-db.org) with a confidence score threshold >0.4.^36^ The PPI network was constructed and visualized using Cytoscape v3.10.3 (https://cytoscape.org/).^37^ Hub proteins were identified based on node degree centrality. Pearson correlation analysis was performed using the corrplot package to evaluate relationships between hub proteins and enriched pathways.

### 2.5 Establishment of a Risk Score Model

#### 2.5.1 Feature Selection and Model Construction

Statistical analysis of proteomic data was performed using RStudio (R Foundation for Statistical Computing, Vienna, Austria). The LASSO method, implemented via the glmnet package, was applied for feature selection and high-dimensional regression analysis. To optimize model performance and prevent overfitting, 10-fold cross-validation was employed during model training, with the lambda (*λ*) parameter set to one standard error of the minimum criterion.^38^ The explicit solution of the LASSO estimator is

$$LASSO\;Regression:\sum_{i=1}^{N}{\left(y_{i}-\sum_{j=1}^{p}x_{ij}\beta_{j}\right)}^{2}+\lambda \sum_{j=1}^{p}\left|\beta_{j}\right|$$

*N* is the sample size, *p* is the number of features, *y*_*i*_ is the observed value for the *i*-th sample, *x*_*ij*_ is the observed value of the *j*-th feature for the *i*-th sample, *β*_*j*_ is the coefficient of the *j*-th feature, and *λ* is the L1 regularization parameter controlling the degree of sparsity.

#### 2.5.2 Correlation Analysis and Predictor Identification

Pearson correlation analysis, conducted using the corrplot package, was performed to evaluate relationships between individual plasma biomarkers and complement and coagulation cascades. Features selected through the LASSO regression were further evaluated using correlation analysis to identify the most robust predictors for the risk model.

#### 2.5.3 Risk Score Calculation

The final risk prediction model for VOCs exposure was developed based on the coefficients derived from LASSO regression. The risk score was calculated using the following formula: risk score = (0.8702836 × VWF) + (0.01839156 × CDC42), where VWF and CDC42 represent the protein expression levels of von Willebrand factor and cell division cycle 42, respectively.

### 2.6 Enzyme-Linked Immunosorbent Assay

#### 2.6.1 Von Willebrand Factor Quantification

Von Willebrand factor (VWF) concentrations in plasma samples from CON and VOCs groups were determined using a commercial mouse VWF ELISA kit (catalog no. KL11696Mu, Shanghai Kanglang, China) according to the manufacturer’s protocol. Briefly, the standards and pretreated samples were incubated at 37 °C for 90 min. Following washing, biotinylated antimouse VWF antibody working solution was added and incubated for 60 min at 37 °C. After additional washing, enzyme conjugate working solution was added and incubated for 30 min at 37 °C in the dark. Subsequently, 3,3′,5,5′-tetramethylbenzidine (TMB) substrate solution was added and allowed to react for 30 min. The reaction was terminated with a stop solution upon development of a visible color gradient. Absorbance was measured at 450 nm by using a microplate reader. VWF concentrations were calculated by interpolation from the standard curve.

#### 2.6.2 Cell Division Cycle 42 Quantification

Cell division cycle 42 (CDC42) levels in plasma samples from CON and VOCs groups were measured using a commercial mouse CDC42 ELISA kit (catalog no. LX-W8454, Wuhan Lianxing, China) following the manufacturer’s instructions. Standards and pretreated samples were incubated with a horseradish peroxidase (HRP) conjugate at 37 °C for 60 min. After washing, the substrate solution was added and incubated for 15 min at 37 °C in the dark. The reaction was terminated with stop solution, and the absorbance was measured at 450 nm within 15 min using a microplate reader. CDC42 concentrations were determined by interpolation from the standard curve.

### 2.7 Statistical Analysis

Statistical analysis was performed using R software (version 4.4.2). Data are presented as the mean ± standard deviation (SD) from at least three biologically independent experiments, each conducted in triplicate. Statistical significance was determined by two-tailed Student’s *t*-test. *P* < 0.05 was considered statistically significant.

## 3 Result

### 3.1 VOCs Exposure Disrupts Hemostatic Balance Characterized by the Dysregulation of Complement and Coagulation Cascades

To investigate the potential impact of VOCs on hemostatic homeostasis, we performed quantitative proteomic profiling of murine plasma samples. Comprehensive quality control assessments confirmed high data quality, with an appropriate peptide length distribution, sufficient unique peptides, adequate protein coverage, and normal molecular weight and isoelectric point distributions (Figure S1A–D). The analytical reliability was further validated by uniform expression patterns across samples, an absence of outliers, and high intersample correlation coefficients (Figure S1E,F). PCA revealed distinct segregation between the VOCs and CON groups, indicating significant proteomic alterations induced by VOCs exposure (Figure 1A). Differentially expressed analysis (|log_2_FC| > 0.263 and *P* < 0.05) identified 439 DEPs, comprising 141 upregulated and 298 downregulated proteins in the VOCs group relative to controls (Figure 1B). Hierarchical clustering analysis demonstrated that these DEPs segregated into two major expression patterns corresponding to upregulation and downregulation (Figure S2).

**Figure 1:**
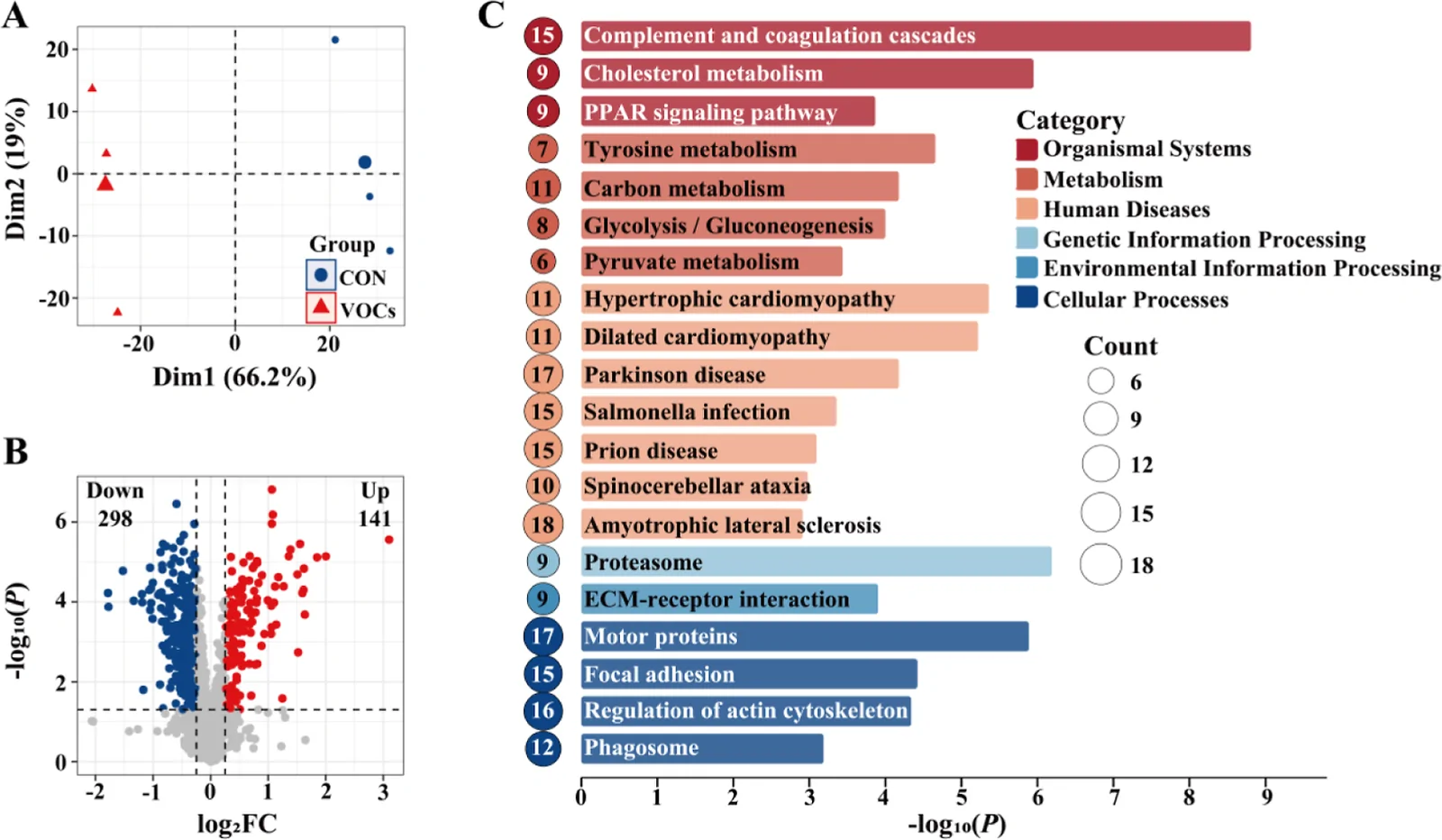
Proteomic alterations in response to VOCs exposure. (A) Comparative analysis of global proteomic profiles between the CON and VOCs groups. (B) Volcano plot illustrating DEPs between the CON and VOCs groups, depicting the relationship between fold-change (*x*-axis) and −Log_10_(*P*) (*y*-axis). (C) KEGG pathway enrichment analysis of DEPs. The *x*-axis represents −log_10_(*P*), and the dot size corresponds to the number of enriched genes within each pathway.

Pathway enrichment analysis showed that the “Complement and Coagulation Cascades” pathway exhibited the most significant enrichment (Figures 1C and S3). This finding was corroborated by Reactome pathway analysis, which indicated significant enrichment of multiple interconnected pathways, including “Intrinsic Pathway of Fibrin Clot Formation”, “Lectin Pathway of Complement Activation”, and “Neutrophil Degranulation” (Table S1). Subcellular localization analysis demonstrated that these DEPs were primarily distributed in the cytoplasm, extracellular region, and membranes (Figure S4). These findings collectively demonstrate that VOCs exposure induces substantial perturbations in plasma proteomic profiles, with the most pronounced alterations occurring in complement and coagulation cascades, suggesting the potential disruption of hemostatic balance.

### 3.2 VOCs Exposure Induces Suppression of Complement and Coagulation Cascades with Potential Pathophysiological Implications

To investigate the regulatory status of the complement and coagulation cascades following VOCs exposure, we performed a GSEA based on the KEGG database. The GSEA results indicated an overall inhibitory state of these pathways postexposure (Figure 2A, Table S2). Hierarchical clustering analysis further confirmed that the majority of DEPs within the cascades exhibited downregulated expression patterns (Figure 2B), collectively indicating a broad suppressive effect of VOCs on complement and coagulation systems.

**Figure 2:**
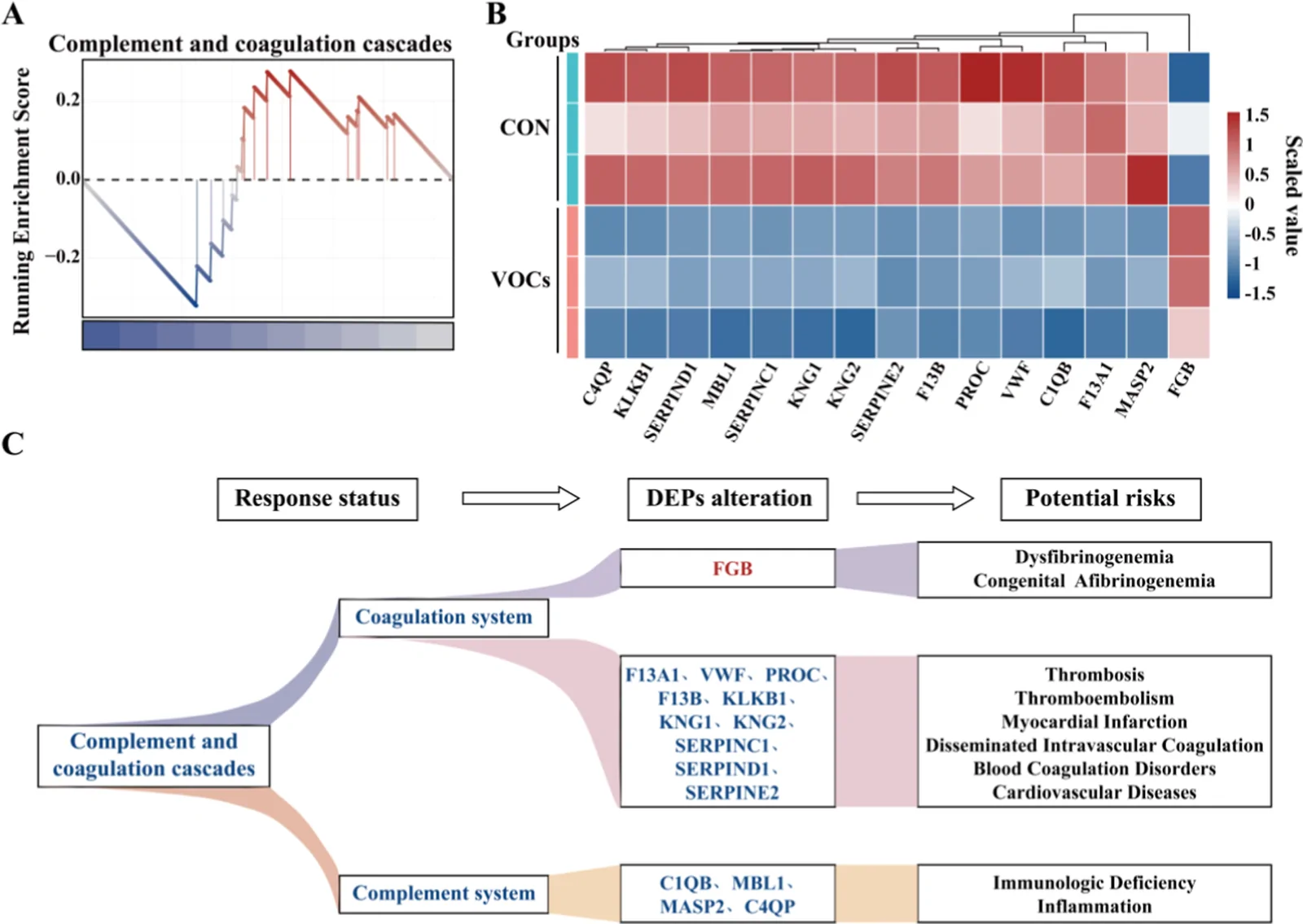
Characterization of complement and coagulation cascades alterations and associated risks. (A) GSEA plot of the complement and coagulation cascades. (B) Heatmap of hierarchical clustering analysis of differentially expressed proteins within the complement and coagulation cascades. (C) Sankey diagram illustrating the potential health effects associated with dysregulation of the complement and coagulation cascades. Blue and red denote downregulated and upregulated states, respectively, while black indicates risk.

Pathway mapping analysis identified specific downregulated proteins critical for hemostatic and immune functions, including coagulation factors (VWF, SERPINC1, F13) and complement components (C1Q, C4B, MASP2) (Figure S5). To elucidate potential health implications, we queried the Comparative Toxicogenomics Database (CTD) for disease associations linked to these dysregulated pathways. The analysis revealed that coordinated suppression of these key proteins may predispose individuals to coagulation disorders (e.g., thrombosis, disseminated intravascular coagulation), immune dysfunction (e.g., immunodeficiency states, inflammatory conditions), and increased cardiovascular risk through a multitarget mechanism (Figure 2C). These findings demonstrate that VOCs exposure induces systemic suppression of complement and coagulation cascades via the downregulation of critical proteins, suggesting potential pathophysiological consequences for hemostatic balance and immune competence.

### 3.3 VOCs Exposure Modulates Complement and Coagulation Cascades through Perturbation of Interconnected Pathways

To elucidate the regulatory networks influencing complement and coagulation cascades following VOCs exposure, we performed functional enrichment network analyses on DEPs, excluding those directly involved in the cascades. Downregulated proteins were significantly enriched in the Rap1 signaling pathway, platelet activation, PI3K-Akt signaling pathway, focal adhesion, and ECM-receptor interaction (Figures 3A and S6A). Conversely, upregulated proteins showed significant enrichment in necroptosis, the IL-17 signaling pathway, and antigen processing and presentation (Figures 3B and S6B).

**Figure 3:**
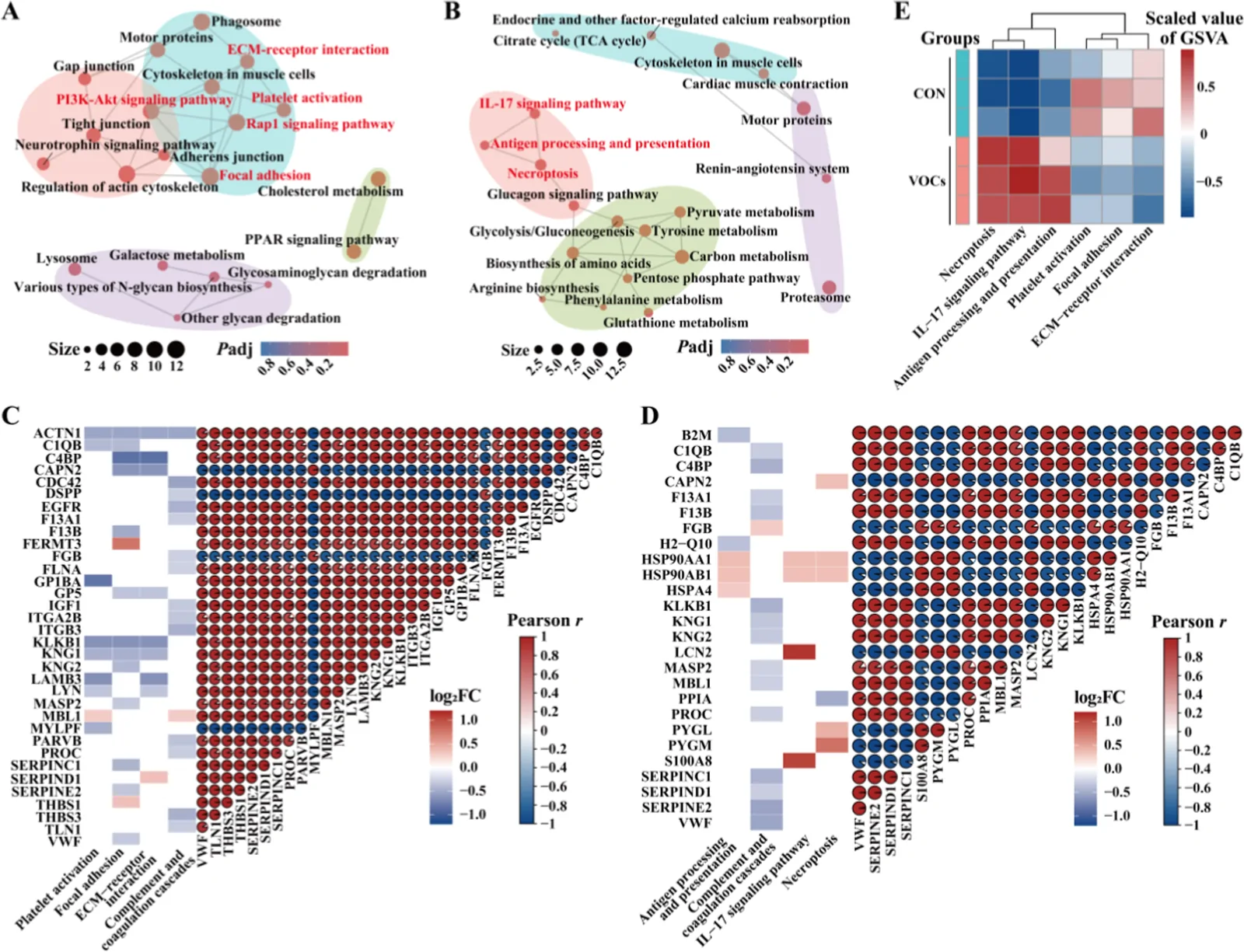
Key pathways perturbing the complement and coagulation cascades and their regulatory states. (A) Network enrichment analysis of remaining downregulated proteins after excluding DEPs in the complement and coagulation cascades. (B) Network enrichment analysis of remaining upregulated proteins after excluding DEPs in the complement and coagulation cascades. (C) Correlation analysis between DEPs in the key pathways of downregulated proteins and DEPs in the complement and coagulation cascades. (D) Correlation analysis between DEPs in the enriched pathways of upregulated proteins and DEPs in the complement and coagulation cascades. (E) Heatmap of GSVA analysis for key pathways.

To identify key pathways potentially modulating complement and coagulation cascades, we performed a correlation analysis between proteins in these pathways and those in the complement and coagulation cascades. Platelet activation, focal adhesion, and ECM–receptor interaction exhibited strong positive correlations with complement and coagulation cascades (Figures 3C and S7). Similarly, necroptosis, the IL-17 signaling pathway, and antigen processing and presentation also demonstrated significant associations with the cascades (Figure 3D). GSVA analysis further validated these findings. Upon VOCs exposure, platelet activation, focal adhesion, and ECM–receptor interaction were suppressed (Figures 3E and S8A), while necroptosis, the IL-17 signaling pathway, and antigen processing and presentation were activated (Figures 3E and S8B). These findings indicate that VOCs exposure directly disrupts complement and coagulation cascades. Additionally, it indirectly modulates their function via coordinated perturbations in interconnected pathways, particularly platelet activation, ECM–receptor interaction, and the IL-17 signaling pathway.

### 3.4 VWF Functions as a Central Mediator in VOCs-Induced Dysregulation of Complement and Coagulation Cascades through Multi-pathway Interactions

To elucidate the molecular interconnections between complement and coagulation cascades and their associated pathways, we conducted a comprehensive analysis using multiple bioinformatics approaches. Sankey diagram analysis identified VWF as the most extensively enriched molecule across multiple pathways, including focal adhesion, complement and coagulation cascades, platelet activation, and ECM–receptor interaction (Figure 4A). To further identify core effector molecules, we constructed a PPI network using the STRING database, which comprised 43 nodes and 190 edges representing interactions among differentially expressed proteins from the seven pathways. Network topology analysis revealed that VWF exhibited the highest degree value (degree score = 19) among all proteins (Figure 4B, Table S3), suggesting its central role in the molecular network.

**Figure 4:**
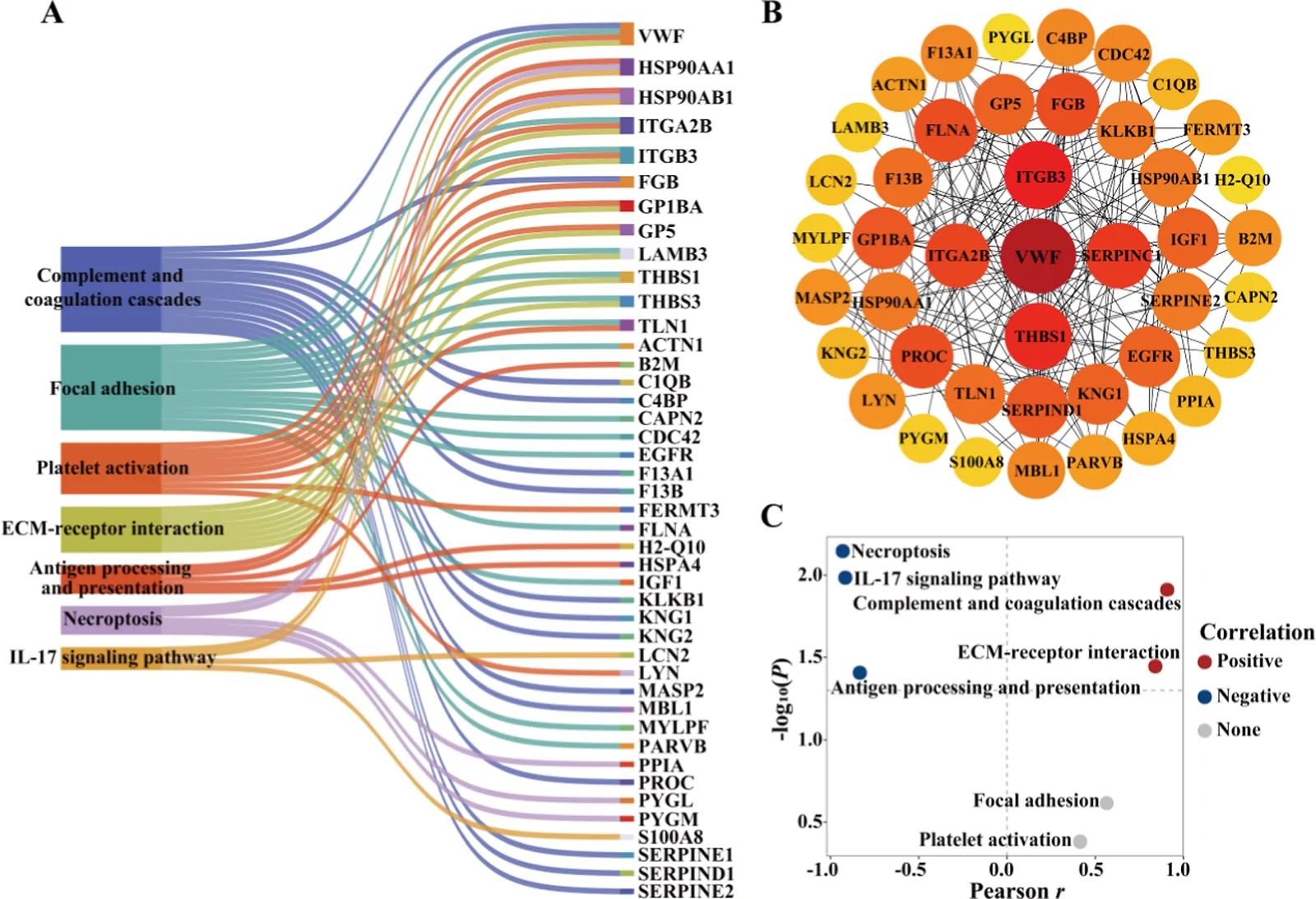
Identification of core effector molecules. (A) Sankey diagram illustrating the involvement of differentially expressed proteins in key pathways and the complement and coagulation cascades. (B) PPI network analysis of differentially expressed proteins within key pathways and the complement and coagulation cascades. Proteins are represented as nodes, and interactions between proteins are depicted as edges. Node color and size correspond to the degree value of each protein. Redder nodes indicate higher rankings, and core effector molecules are highlighted in crimson. (C) Scatter plot showing the correlation between VWF and key pathways, as well as the complement and coagulation cascades.

Correlation analysis further substantiated VWF’s pivotal position, demonstrating significant positive correlations with complement and coagulation cascades (*r* = 0.908, *P* = 0.012) and ECM–receptor interaction (*r* = 0.841, *P* = 0.036). Conversely, VWF showed significant negative correlations with antigen processing and presentation (*r* = −0.834, *P* = 0.039), the IL-17 signaling pathway (*r* = −0.916, *P* = 0.010), and necroptosis (*r* = −0.930, *P* = 0.007). No significant correlations were observed with focal adhesion or platelet activation (Figure 4C, Table S4). These findings establish VWF as a key effector in the VOCs-induced disruption of complement and coagulation cascades. Its broad interactions across related pathways suggest a central role in the molecular mechanisms of VOCs toxicity.

### 3.5 Development and Validation of a Predictive Risk Score Model for VOCs-Induced Dysregulation of Complement and Coagulation Cascades

To establish a predictive risk score model for the dysregulation of complement and coagulation cascades following VOCs exposure, we systematically identified potential biomarkers through machine learning. Using LASSO regression analysis with variable shrinkage and cross-validation, we identified four candidate biomarkers—VWF, Fibrinogen Beta Chain (FGB), CDC42, and Glycogen Phosphorylase L (PYGL)—from differentially expressed proteins within critical pathways (Figures 5A and S9, Table S5). Subsequent correlation analysis revealed that VWF and CDC42 exhibited the strongest associations with complement and coagulation cascades and the interconnected pathways (Figures 5B and S10), indicating their central roles in mediating VOCs-induced cascade dysregulation. Based on these findings, we constructed a weighted risk score model using coefficients derived from LASSO regression analysis. The optimal penalty coefficient (*λ*) was determined via 10-fold cross-validation, where an increasing *λ* value led to a gradual reduction in the number of variables retained in the model. The *λ* value corresponding to the minimum cross-validation error (*λ* = 0.018281) was selected for the final model (Figure 5C). At this *λ*, the final model incorporated two variables, VWF and CDC42, which were assigned weight coefficients of 0.8702836 and 0.01839156, respectively (Figure 5D, Table S5). Risk score evaluation demonstrated a significant decrease following VOCs exposure (*P* < 0.05), establishing that lower scores correspond to higher risk of cascade dysregulation (Figure 5E, Table S6).

**Figure 5:**
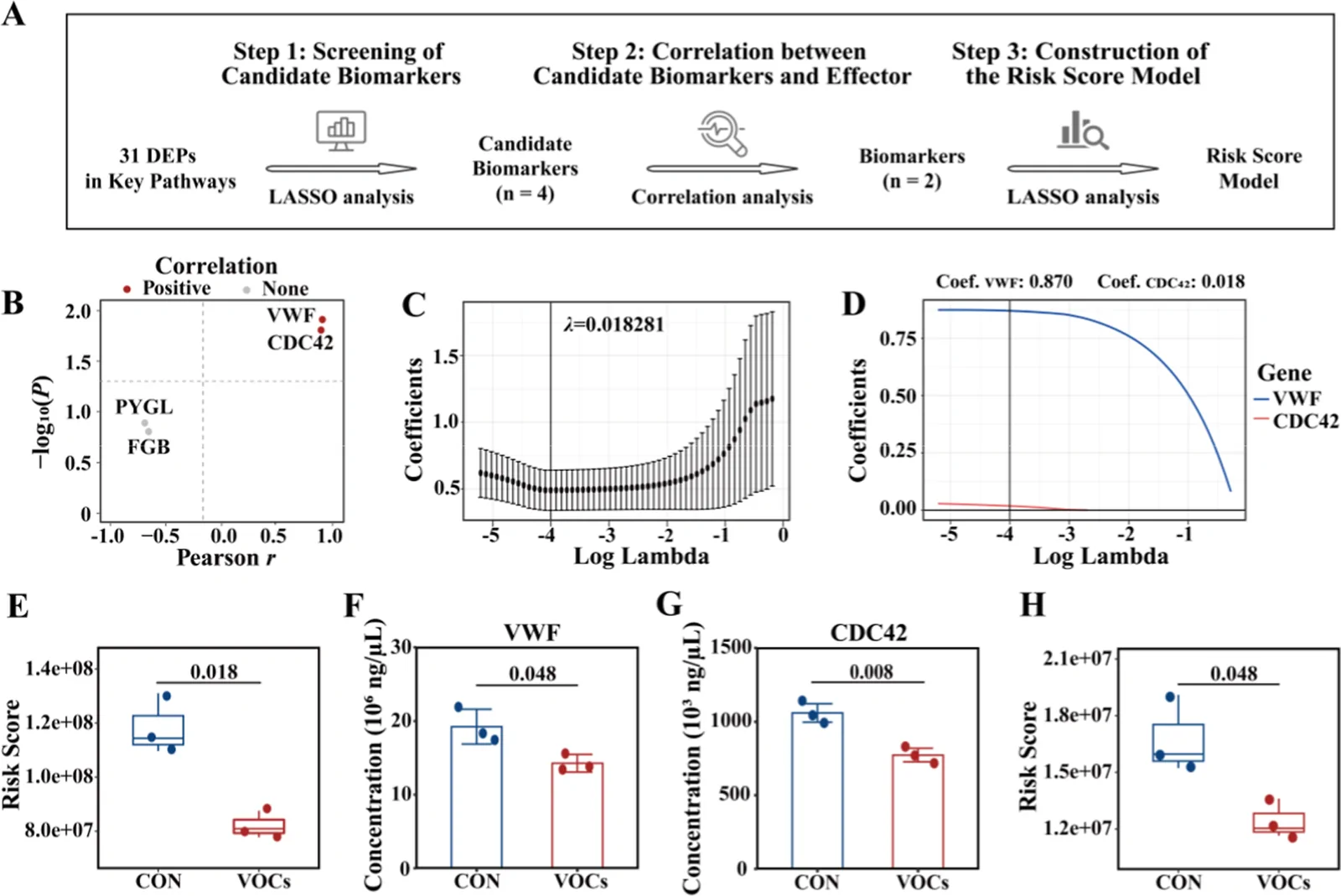
Construction and validation of the risk score model. (A) Flowchart of the risk score model construction process. (B) Correlation analysis between the four identified biomarkers and the complement and coagulation cascades. (C) Selection of the tuning parameter (*λ*) in the LASSO model using 10-fold cross-validation based on the minimum criterion. (D) Variation of the coefficients for two selected markers with respect to the optimal parameter (*λ*). (E) Comparison of risk scores based on proteomic data. (F) Expression level of VWF in mouse plasma. The range of the error bars is mean ± SD. (G) Expression level of CDC42 in mouse plasma. The range of the error bars is mean ± SD. (H) Comparison of risk scores based on experimental validation data.

Experimental validation confirmed significantly decreased plasma levels of both VWF (*P* < 0.05; Figure 5F, Table S7) and CDC42 (*P* < 0.05; Figure 5G, Table S7) in the VOCs group, consistent with model predictions. Application of experimentally determined values to the model effectively discriminated between CON and VOCs groups (Figure 5H, Table S8), demonstrating its robustness and clinical applicability. In conclusion, we developed and validated a predictive risk score model based on VWF and CDC42 expression for the dysregulation of assessing complement and coagulation cascades following VOCs exposure. This model provides a valuable tool for future investigations and the potential clinical risk assessment in VOCs-exposed populations.

## 4 Discussion

Indoor air pollution poses a significant public health challenge, particularly concerning the health risks associated with VOCs from indoor renovation materials.^2^ While traditional exposure assessment methods have limitations in capturing actual biological effects,^15^ direct biomarker identification in exposed populations offers a more precise approach to risk evaluation.^39^ To address this critical knowledge gap, our study employed a whole-body inhalation exposure mouse model to comprehensively investigate the proteomic alterations in plasma following VOCs exposure. A key innovation of our research lies in the systematic characterization of proteomic changes, which enabled us to identify core effect biomarkers and develop a novel risk score model with high discriminative capability. Our findings reveal that VOCs exposure significantly disrupts the complement and coagulation cascades, potentially leading to impaired innate immunity and coagulation homeostasis. This core pathway disturbance is critically linked to six additional pathways, including platelet activation and the IL-17 signaling pathway. Importantly, we identify VWF and CDC42 as potential biomarkers for VOCs-induced hematological effects and develop a risk score model with high discriminative capability. Our findings clarify the molecular signature of VOCs-induced blood homeostasis imbalance and identify practical biomarkers for risk assessment, thereby providing a novel foundation for preventing and mitigating related health effects.

While the multisystem toxicity of VOCs is well-established, their specific impact on the complement and coagulation systems under realistic exposure scenarios remains poorly understood.^15,16,40–42^ Previous investigations have been largely confined to studying single VOCs constituent or employing unrealistically high exposure concentrations, which fail to capture the complex proteomic alterations resulting from real-world, multicomponent exposure.^22,23^ To bridge this translational gap, we employed a physiologically relevant whole-body inhalation mouse model that closely simulates actual environmental exposure. A key and novel finding from our high-throughput proteomic analysis is the significant and coordinated downregulation of proteins within both the complement and coagulation cascades (Figure 1), revealing a systemic inhibitory trend (Figure 2A,B). This observation aligns with, yet critically extends, prior reports linking specific VOCs, such as benzene homologues, to isolated instances of coagulopathy and reduced complement component levels (e.g., C3, C4).^43,44^ Importantly, our data move beyond these component-specific findings to suggest a more comprehensive mechanism. We propose that realistic, multicomponent VOCs exposure may suppress the complement and coagulation cascades. This suppression could occur by directly disrupting their functions or by depleting relevant proteins through immunosuppression and sustained chronic inflammation.

The complement and coagulation systems are integral to host defense and hemostatic balance, and their dysregulation is a recognized pathogenic driver in numerous diseases.^45–47^ Our proteomic analysis revealed significant perturbations in these cascades following VOCs exposure. To translate these molecular findings into a clinical context, we performed a systematic analysis using the CTDbase. This analysis projected that the observed perturbations could substantially elevate the risk of a spectrum of severe pathologies, including thrombosis, myocardial infarction, disseminated intravascular coagulation (DIC), and immunodeficiency (Figure 2C). These bioinformatic predictions are strongly supported by established pathophysiological evidence. Dysregulation of the complement system is known to compromise host defense and inflammatory control, predisposing individuals to infections and immunodeficiency.^48,49^ Likewise, coagulation abnormalities are linked to DIC, thrombotic events, and cardiovascular disorders, findings that align precisely with our CTDbase-based risk projections.^47,50–53^ Critically, the complement and coagulation systems are not isolated pathways but engage in extensive cross-talk (Figure S5), forming a highly interdependent network where inhibition of one system can profoundly influence the other, creating a precarious and dynamic equilibrium. For instance, complement inhibition can dampen platelet activation and thrombin generation, while coagulation dysfunction can conversely impact immune responses and increase bleeding risk.^54^ Therefore, our study provides a crucial bridge, linking an environmental exposure (VOCs) to a specific molecular signature (complement and coagulation perturbations) and consequently to a defined spectrum of clinical risks. By establishing this link, our findings lay a critical theoretical foundation for developing targeted risk prediction models and preventive strategies for VOCs-associated cardiovascular and immune disorders.

Prior studies have established an association between environmental pollutants and dysregulation of the complement and coagulation systems.^55,56^ Nevertheless, the specific relationships between individual VOCs and these pathways remain to be fully elucidated. Our findings demonstrate that VOCs exposure is associated with coordinated alterations across multiple related pathways, suggesting potential system-level involvement that warrants further mechanistic investigation. We identified several key signaling pathways—focal adhesion, platelet activation, the IL-17 signaling pathway, ECM–receptor interaction, apoptosis, and antigen processing and presentation—that were significantly modulated upon exposure to VOCs (Figure 3). These pathways converge on the complement and coagulation systems through distinct yet interconnected mechanisms. For instance, the focal adhesion and ECM–receptor interaction regulate platelet adhesion and activation, a central event in thrombus formation.^57,58^ Concurrently, the IL-17 signaling pathway, a potent driver of inflammation, can directly modulate the expression of both coagulation factors and complement components, linking inflammation to thrombosis.^59,60^ Furthermore, apoptosis and antigen processing and presentation contribute to this network by releasing immunomodulatory molecules and forming complexes that can further influence immune homeostasis and, by extension, coagulation balance.^61,62^ This finding suggests that exposure to VOCs may collectively influence the complement and coagulation cascades within a broader regulatory network via synergistic actions of multiple pathways.

While risk score models are well-established in fields like oncology for prognosis and recurrence prediction, their application in assessing health risks from real-world, complex environmental exposures such as VOCs remains nascent.^63,64^ To bridge this translational gap, we employed LASSO regression, a powerful method renowned for its ability to distill the most predictive features from high-dimensional data while preventing overfitting, thereby enhancing both the model accuracy and interpretability. Through this approach, we identified two key biomarkers, VWF and CDC42.^65^ For instance, VWF can serve as a biomarker for assessing the risk of thrombosis, while CDC42 can act as a prognostic risk biomarker for colorectal cancer.^66,67^ Based on the two key biomarkers, we also constructed a robust risk score model to quantitatively assess the perturbation of the complement and coagulation systems following VOCs exposure (Figure 5). The model’s coefficients, weighted by the expression levels of these proteins, provide a direct measure of their relative contribution to the overall risk. It enables quantitative, individual risk assessment for diverse VOCs exposures through a simple blood test, supporting early detection and targeted intervention.

## 5 Environmental Implication

This study integrates an indoor-realistic VOCs exposure model, high-throughput proteomics, and machine learning to reveal, for the first time, the proteomic signatures of VOCs-induced disruption to hemostatic balance, mainly characterized by the abnormal response of complement and coagulation. We identified VWF and CDC42 as key biomarkers and developed a robust risk score model, providing a novel framework for quantifying individual health risks from complex environmental exposures. Our findings demonstrate that common indoor VOCs mixtures significantly perturb these critical physiological pathways, underscoring the need for stricter regulations on building materials and indoor air quality to protect public health. While our findings provided foundational insights, future work incorporating chronic animal models and human cohort studies is warranted to validate dose–responses, refine the model’s predictive power, and ultimately inform targeted interventions for susceptible populations. This integrated strategy bridges proteomics-driven disruption of hemostatic balance with the risk assessment of VOCs, advancing the field of environmental health sciences and offering a blueprint for evaluating other complex pollutants.

## Supplementary Material



## Data Availability

The data that support this study are available within the article and its Supporting Information files or available from the upon request.

## Notes

Data quality control in proteomic analysis (Figure S1); heatmap of KEGG pathway enrichment cluster analysis for DEPs between the CON and VOCs groups (Figure S2); KEGG pathway enrichment analysis of DEPs between the CON and VOCs groups (Figure S3); subcellular localization of DEPs between the CON and VOCs groups (Figure S4); potential mechanisms underlying alterations in the complement and coagulation cascades following VOCs exposure (Figure S5); enrichment analysis of the remaining differentially expressed proteins after excluding those in the complement and coagulation cascades (Figure S6); correlation analysis between DEPs in the enriched pathways of downregulated proteins and DEPs in the complement and coagulation cascade (Figure S7); alteration profiles of differentially expressed proteins in key pathways (Figure S8); LASSO analysis of differentially expressed proteins in the six key pathways (Figure S9); correlation analysis of candidate biomarkers with the complement and coagulation cascades and six key pathways (Figure S10); Reactome pathway analysis of DEPs between the CON and VOCs groups (Table S1); GSEA of the complement and coagulation cascades (Table S2); ranking of degree values for DEPs in key pathways and the complement and coagulation cascades (Table S3); correlation analysis of VWF with the complement and coagulation cascades and key pathways (Table S4); LASSO model output results (Table S5); risk scores of the risk score model (Table S6); protein expression of VWF and CDC42 (Table S7); and risk scores for validating the risk score model (Table S8) (PDF)

## References

[1] The Institute for Health Metrics and Evaluation . Air Pollution. https://www.healthdata.org/research-analysis/health-topics/air-pollution.

[2] Global burden and strength of evidence for 88 risk factors in 204 countries and 811 subnational locations, 1990–2021: a systematic analysis for the Global Burden of Disease Study 2021. Lancet 2024, 403 (10440), 2162–2203. DOI: 10.1016/s0140-6736(24)00933-4. 38762324 PMC11120204

[3] Paterson C. A., Sharpe R. A., Taylor T., Morrissey K. (2021). Indoor PM2.5,
VOCs and asthma outcomes: A systematic review in adults and their
home environments. Environ. Res..

[4] Joint Research Centre . Indoor air pollution: new EU research reveals higher risks than previously thought. 2005. https://cordis.europa.eu/article/id/95197-indoor-air-pollution-new-eu-research-reveals-higher-risks-than-previously-thought.

[5] Zheng J., Wu M., Pang Y., Liu Q., Liu Y., Jin X., Tang J., Bao L., Niu Y., Zheng Y. (2024). Interior decorative volatile organic compounds
exposure induces sleep
disorders through aberrant branched chain amino acid transaminase
2 mediated glutamatergic signaling resulting from a neuroinflammatory
cascade. Sci. Total Environ..

[6] Horvat T., Pehnec G., Jakovljević I. (2025). Volatile Organic Compounds in Indoor
Air: Sampling, Determination, Sources, Health Risk, and Regulatory
Insights. Toxics.

[7] Uchiyama S., Tomizawa T., Tokoro A., Aoki M., Hishiki M., Yamada T., Tanaka R., Sakamoto H., Yoshida T., Bekki K. (2015). Gaseous chemical compounds in indoor and outdoor air
of 602 houses throughout Japan in winter and summer. Environ. Res..

[8] You B., Zhou W., Li J., Li Z., Sun Y. (2022). A review of
indoor Gaseous organic compounds and human chemical Exposure: Insights
from Real-time measurements. Environ. Int..

[9] Lunderberg D. M., Misztal P. K., Liu Y., Arata C., Tian Y., Kristensen K., Weber R. J., Nazaroff W. W., Goldstein A. H. (2021). High-Resolution
Exposure Assessment for Volatile Organic Compounds in Two California
Residences. Environ. Sci. Technol..

[10] Yang P., Cheng W., Wang M., Wu M., Liu Q., Zhang J., Pang Y., Niu Y., Tang J., Zhang R. (2025). Ex Vivo Biosensor Strategy Reveals
the Vascular Toxic Effects of
Volatile Organic Compounds Derived from Indoor Renovation. Environ. Health..

[11] Scholten B., Vlaanderen J., Stierum R., Portengen L., Rothman N., Lan Q., Pronk A., Vermeulen R. A. (2020). Quantitative
Meta-Analysis of the Relation between Occupational Benzene Exposure
and Biomarkers of Cytogenetic Damage. Environ.
Health Perspect..

[12] Liu N., Bu Z., Liu W., Kan H., Zhao Z., Deng F., Huang C., Zhao B., Zeng X., Sun Y. (2022). Health effects of exposure
to indoor volatile organic compounds from
1980 to 2017: A systematic review and meta-analysis. Indoor Air.

[13] Yu H., Zhang J., Liu Q., Li D., Pan R., Miao G., Chen Y., Kou Z., Qu G., Zhang R. (2026). Interior Decorative VOCs Elevate T Cell-Mediated Obstructive
Lung Disease Risks via Osteogenesis-Driven Lymphoid-Biased Hematopoiesis. Adv. Sci.

[14] McGraw K. E., Domingo-Relloso A., Riggs D. W., Medgyesi D. N., Neupane R., Stingone J. A., Sanchez T. R. (2025). Exposure to Volatile Organic Compounds
and Blood Pressure in NHANES 2011 to 2018. Hypertension.

[15] Miao G., Wang Y., Wang B., Yu H., Liu J., Pan R., Zhou C., Ning J., Zheng Y., Zhang R. (2023). Multi-omics analysis
reveals hepatic lipid metabolism profiles and
serum lipid biomarkers upon indoor relevant VOC exposure. Environ. Int..

[16] Liu Q., Niu Y., Pei Z., Yang Y., Xie Y., Wang M., Wang J., Wu M., Zheng J., Yang P. (2024). Gas6-Axl signal promotes
indoor VOCs exposure-induced pulmonary fibrosis
via pulmonary microvascular endothelial cells-fibroblasts cross-talk. J. Hazard. Mater..

[17] Geyer P. E., Holdt L. M., Teupser D., Mann M. (2017). Revisiting biomarker
discovery by plasma proteomics. Mol. Syst. Biol..

[18] Wallace M. A., Kormos T. M., Pleil J. D. (2016). Blood-borne biomarkers
and bioindicators
for linking exposure to health effects in environmental health science. J. Toxicol. Environ. Health B Crit. Rev..

[19] Avogbe P. H., Ayi-Fanou L., Autrup H., Loft S., Fayomi B., Sanni A., Vinzents P., Møller P. (2005). Ultrafine
particulate matter and high-level benzene urban air pollution in relation
to oxidative DNA damage. Carcinogenesis.

[20] Bolden A. L., Kwiatkowski C. F., Colborn T. (2015). New Look at BTEX: Are Ambient Levels
a Problem?. Environ. Sci. Technol..

[21] Manisalidis I., Stavropoulou E., Stavropoulos A., Bezirtzoglou E. (2020). Environmental
and Health Impacts of Air Pollution: A Review. Front. Public Health.

[22] Davidson C. J., Hannigan J. H., Bowen S. E. (2021). Effects
of inhaled combined Benzene,
Toluene, Ethylbenzene, and Xylenes (BTEX): Toward an environmental
exposure model. Environ. Toxicol. Pharmacol..

[23] Scofield S., Koshko L., Stilgenbauer L., Booms A., Berube R., Kassotis C., Lin C. H., Jang H., Kim S., Stemmer P. (2025). Integrative
multi-omics analysis of metabolic dysregulation
induced by occupational benzene exposure in mice. Sci. Total Environ..

[24] Ye C., Xia L., Gong R., Chang J., Sun Q., Xu J., Li F. (2025). Integrating
plasma proteome with genome reveals novel protein biomarkers
in colorectal cancer. Clin. Transl. Oncol..

[25] Ferkingstad E., Sulem P., Atlason B. A., Sveinbjornsson G., Magnusson M. I., Styrmisdottir E. L., Gunnarsdottir K., Helgason A., Oddsson A., Halldorsson B. V. (2021). Large-scale integration of the plasma proteome with genetics and
disease. Nat. Genet..

[26] Shi C., Yang X., Liu J., Tian H., Gao J., Yan Y., Peng G., Qin H., Lv M., Liu Y. (2025). Nontargeted Toxicological/Chemical
Analysis in Complex Mixtures for
Risk Assessment and Key Driver Discovery. Environ.
Health..

[27] Deng Y. T., You J., He Y., Zhang Y., Li H. Y., Wu X. R., Cheng J. Y., Guo Y., Long Z. W., Chen Y. L. (2025). Atlas of the plasma
proteome in health and disease in 53,026 adults. Cell.

[28] Zhang Z., Wu S., Stenoien D. L., Paša-Tolić L. (2014). High-throughput proteomics. Annu. Rev. Anal Chem. (Palo Alto Calif).

[29] Mohanty B. P., Mahanty A., Mitra T., Mohanty S., Naik A. K., Parija S. C. (2020). Proteomic and transcriptomic
changes in rat liver following
oral feeding of formaldehyde. Chemosphere.

[30] Declercq A., Devreese R., Scheid J., Jachmann C., Van Den
Bossche T., Preikschat A., Gomez-Zepeda D., Rijal J. B., Hirschler A., Krieger J. R. (2025). TIMS­(2)­Rescore:
A Data Dependent Acquisition-Parallel Accumulation and Serial Fragmentation-Optimized
Data-Driven Rescoring Pipeline Based on MS(2)­Rescore. J. Proteome Res..

[31] Zhang W., Guo X., Ren J., Chen Y., Wang J., Gao A. (2022). GCN5-mediated
PKM2 acetylation participates in benzene-induced hematotoxicity through
regulating glycolysis and inflammation via p-Stat3/IL17A axis. Environ. Pollut..

[32] Chen J. C., Goodrich J. A., Walker D. I., Liao J., Costello E., Alderete T. L., Valvi D., Hampson H., Li S., Baumert B. O. (2024). Exposure
to per- and polyfluoroalkyl substances
and high-throughput proteomics in Hispanic youth. Environ. Int..

[33] Miao G., Zhou C., Xu L., Zhao L., Zhang J., Zhang Z., Kou Z., Ahmed R. Z., Lu D., Jin X. (2025). Evaluating
the Vascular Risk of PFCs: An Integrated
XGBoost-Driven Structure-Activity Prediction and Experimental Validation
Study. Environ. Health..

[34] He X., Zhang J., Gong M., Gu Y., Dong B., Pang X., Zhang C., Cui Y. (2023). Identification
of potential
ferroptosis-associated biomarkers in rheumatoid arthritis. Front. Immunol..

[35] Hänzelmann S., Castelo R., Guinney J. (2013). GSVA: gene
set variation analysis
for microarray and RNA-seq data. BMC Bioinf..

[36] Li P., Fei C. S., Chen Y. L., Chen Z. S., Lai Z. M., Tan R. Q., Yu Y. P., Xiang X., Dong J. L., Zhang J. X. (2022). Revealing the novel autophagy-related genes
for ligamentum flavum hypertrophy in patients and mice model. Front. Immunol..

[37] Li Y., Yao Q., Xu H., Ren J., Zhu Y., Guo C., Li Y. (2024). Lung Single-Cell Transcriptomics
Offers Insights into the Pulmonary
Interstitial Toxicity Caused by Silica Nanoparticles. Environ. Health..

[38] Zhang Y., Zhao S., Cao W., Sun S., Zeng Q., Luo P. (2024). Ambient Ozone Exposure, Semen Plasma
Metabolites, and Sperm Quality
Decline among Adult Men in Wuhan China. Environ.
Health..

[39] Ho F. K., Mark P. B., Lees J. S., Pell J. P., Strawbridge R. J., Kimenai D. M., Mills N. L., Woodward M., McMurray J. J. V., Sattar N. (2025). A Proteomics-Based
Approach for Prediction
of Different Cardiovascular Diseases and Dementia. Circulation.

[40] Cao Q., Liu J., Lu D., Song J., Yu Q., Wen J., Zheng B. (2025). Dissecting
the connection between volatile organic compounds and
cardiovascular disease: immune cell-mediated mechanism. Ecotoxicol. Environ. Saf..

[41] Ji W., Wang Y., Zhao B., Liu J. (2024). Identifying high-risk
volatile organic compounds in residences of Chinese megacities: A
comprehensive health-risk assessment. J. Hazard.
Mater..

[42] Fujimaki H., Yamamoto S., Tin Tin
Win S., Hojo R., Sato F., Kunugita N., Arashidani K. (2007). Effect of
long-term exposure to low-level
toluene on airway inflammatory response in mice. Toxicol. Lett..

[43] Tongsantia U., Chaiklieng S., Suggaravetsiri P., Andajani S., Autrup H. (2021). Factors Affecting
Adverse Health Effects of Gasoline Station Workers. Int. J. Environ. Res. Public Health.

[44] Sauer E., Gauer B., Nascimento S., Nardi J., Göethel G., Costa B., Correia D., Matte U., Charão M., Arbo M. (2018). The role of B7 costimulation in benzene immunotoxicity
and its potential association with cancer risk. Environ. Res..

[45] Dobó J., Kocsis A., Farkas B., Demeter F., Cervenak L., Gál P. (2024). The Lectin
Pathway of the Complement System-Activation,
Regulation, Disease Connections and Interplay with Other (Proteolytic)
Systems. Int. J. Mol. Sci..

[46] Garred P., Tenner A. J., Mollnes T. E. (2021). Therapeutic Targeting of the Complement
System: From Rare Diseases to Pandemics. Pharmacol.
Rev..

[47] Wilhelm G., Mertowska P., Mertowski S., Przysucha A., Strużyna J., Grywalska E., Torres K. (2023). The Crossroads of the
Coagulation System and the Immune System: Interactions and Connections. Int. J. Mol. Sci..

[48] McMurray J. C., Schornack B. J., Weskamp A. L., Park K. J., Pollock J. D., Day W. G., Brockshus A. T., Beakes D. E., Schwartz D. J., Mikita C. P. (2024). Immunodeficiency: Complement disorders. Allergy Asthma Proc..

[49] Leonardi L., La Torre F., Soresina A., Federici S., Cancrini C., Castagnoli R., Cinicola B. L., Corrente S., Giardino G., Lougaris V. (2022). Inherited defects in
the complement system. Pediatr. Allergy Immunol..

[50] Adelborg K., Larsen J. B., Hvas A. M. (2021). Disseminated
intravascular coagulation:
epidemiology, biomarkers, and management. Br.
J. Hamaetol..

[51] Huang S., Ninivaggi M., Chayoua W., de Laat B. V. W. F. (2021). Platelets
and the Antiphospholipid Syndrome. Int. J. Mol.
Sci..

[52] Ji Y., Zhang M. J., Wang W., Norby F. L., Eaton A. A., Inciardi R. M., Alonso A., Sedaghat S., Ganz P., Van’t Hof J. (2025). Association of Coagulation Factor XI Level
With Cardiovascular Events and Cardiac Function in Community-Dwelling
Adults: From ARIC and CHS. Circulation.

[53] Luyendyk J. P., Flick M. J., Wolberg A. S. (2025). Factor
XIII: driving (cross-)­links
in hemostasis, thrombosis, and disease. Blood.

[54] Noris M., Galbusera M. (2023). The complement
alternative pathway and hemostasis. Immunol.
Rev..

[55] Guo Y., Niu S., Zhang Y., Yan W., Ji S., Ma L., Li G., Sang N. (2025). Maternal PM(2.5)
Exposure and Cardiac Developmental
Abnormalities in Fetuses and Neonates: Progressive Activation of the
Complement and Coagulation Cascade. Environ.
Sci. Technol..

[56] Zheng Z., Li H., Zhang Z., Zhai X., Qin H. (2024). Study on the underlying
molecular mechanism of benzene-induced nervous system damage in mice
based on tandem mass tag (TMT) proteomics. Toxicol
Res..

[57] Barreca M. M., Raimondo S., Conigliaro A., Siragusa S., Napolitano M., Alessandro R., Corrado C. (2024). The Combination of Natural Compounds
Escin-Bromelain-Ginkgo Biloba-Sage Miltiorrhiza (EBGS) Reduces Platelet
Adhesion to TNFα-Activated Vascular Endothelium through FAK
Signaling. Int. J. Mol. Sci..

[58] Neubauer K., Zieger B. (2022). Endothelial cells and coagulation. Cell Tissue Res..

[59] Park Y., Shim Y., Kwon I., Lee H. W., Nam H. S., Choi H. J., Heo J. H. (2022). Effects of Interleukin-17A
on the
Early Stages of Arterial Thrombosis in Mice. Yonsei Med. J..

[60] Chen Y., Man-Tak Chu J., Liu J. X., Duan Y. J., Liang Z. K., Zou X., Wei M., Xin W. J., Xu T., Tin-Chun
Wong G. (2025). Double negative T cells promote surgery-induced neuroinflammation,
microglial engulfment and cognitive dysfunction via the IL-17/CEBPβ/C3
pathway in adult mice. Brain, Behav., Immun..

[61] Bertheloot D., Latz E., Franklin B. S. (2021). Necroptosis,
pyroptosis and apoptosis:
an intricate game of cell death. Cell. Mol.
Immunol..

[62] Schriek P., Ching A. C., Moily N. S., Moffat J., Beattie L., Steiner T. M., Hosking L. M., Thurman J. M., Holers V. M., Ishido S. (2022). Marginal zone B cells acquire dendritic cell functions
by trogocytosis. Science.

[63] Wang Y., Wang D. Y., Bu K. N., Gao J. D., Zhang B. L. (2024). Prognosis
prediction and risk stratification of breast cancer patients based
on a mitochondria-related gene signature. Sci.
Rep.

[64] Yan Z. J., Yu C. T., Chen L., Wang H. Y. (2023). Development of a
TMErisk model based on immune infiltration in tumour microenvironment
to predict prognosis of immune checkpoint inhibitor treatment in hepatocellular
carcinoma. Brief Bioinform.

[65] Yin T., Shi S., Zhu X., Cheang I., Lu X., Gao R., Zhang H., Yao W., Zhou Y., Li X. (2022). A Survival
Prediction for Acute Heart Failure Patients via Web-Based Dynamic
Nomogram with Internal Validation: A Prospective Cohort Study. J. Inflamm Res..

[66] Ladikou E. E., Sivaloganathan H., Milne K. M., Arter W. E., Ramasamy R., Saad R., Stoneham S. M., Philips B., Eziefula A. C., Chevassut T. (2020). Von Willebrand factor (vWF): marker of endothelial
damage and thrombotic risk in COVID-19?. Clin
Med..

[67] Zhang D., Tang W., Niu H., Tse W., Ruan H. B., Dolznig H., Knösel T., Karl-Heinz F., Themanns M., Wang J. (2024). Monogenic
deficiency
in murine intestinal Cdc42 leads to mucosal inflammation that induces
crypt dysplasia. Genes Dis..

