# Supplementary material for: Indoor VOCs Exposure Disrupts Hemostatic Balance: Plasma Proteomics Identifies Complement and Coagulation Cascade Dysregulation as a Critical Signature
Source: Environ Health (Wash). 2026 Mar 19;4(8):1625–36. doi: 10.1021/envhealth.5c00641 (PMC13504561; doi:10.1021/envhealth.5c00641)
Supplement: Supplementary file 1 [file eh5c00641_si_001.pdf]

Supplementary Information for

"Indoor VOCs Exposure Disrupts Hemostatic Balance: Plasma Proteomics Identifies Complement and Coagulation Cascade Dysregulation as a Critical Signature"

Yurong Cui[1,#], Gan Miao[1,#], Duxing Li[4], Tian Qiu[1], Peijie Sun[2], Yuebin Lv[2], Xiaohan Sun[1], Yijiao Li[1], Rong Zhang[3], Lin Lu[1,*], Xiaoting Jin[1,*] , Yuxin Zheng[1]

[1] Department of Occupational Health and Environmental Health, School of Public Health, Qingdao University, Qingdao, China
[2] China CDC Key Laboratory of Environment and Population Health, National Institute of Environmental Health, Chinese Center for Disease Control and Prevention, Beijing 100021, China
[3] Department of Toxicology, School of Public Health, Hebei Medical University, Shijiazhuang 050017, China
[4] ZaoZhuang Center for Disease Control and Prevention, Zaozhuang 277101, China

[*] Corresponding author: lulin@qdu.edu.cn (L. Lin); xtjin@qdu.edu.cn (X. Jin).
[#] Contributed equally

Contents

Supplementary Figures

Figure S1. Data Quality Control in Proteomic Analysis.

Figure S2. Heatmap of KEGG Pathway Enrichment Cluster Analysis for DEPs between the CON and VOCs Groups.

Figure S3. KEGG Pathway Enrichment Analysis of DEPs between the CON and VOCs Groups.

Figure S4. Subcellular Localization of DEPs between the CON and VOCs Groups.

Figure S5. Potential Mechanisms Underlying Alterations in the Complement and Coagulation Cascades Following VOCs Exposure.

Figure S6. Enrichment Analysis of the Remaining Differentially Expressed Proteins After Excluding Those in the Complement and Coagulation Cascades.

Figure S7. Correlation Analysis between DEPs in the Enriched Pathways of Downregulated and DEPs in the Complement and Coagulation Cascade.

Figure S8. Alteration Profiles of Differentially Expressed Proteins in Key Pathways.

Figure S9. LASSO Analysis of Differentially Expressed Proteins in the Six Key Pathways.

Figure S10. Correlation Analysis of Candidate Biomarkers with the Complement and Coagulation Cascades and Six Key Pathways.

Supplementary Tables

Table S1. Reactome Pathway Analysis of DEPs between the CON and VOCs Groups.

Table S2. GSEA of the Complement and Coagulation Cascades.

Table S3. Ranking of Degree Values for DEPs in Key Pathways and the Complement and Coagulation Cascades.

Table S4. Correlation Analysis of VWF with the Complement and Coagulation Cascades and Key Pathways.

Table S5. LASSO Model Output Results.

Table S6. Risk Scores of the Risk Score Model.

Table S7. Protein Expression of VWF and CDC42.

Table S8. Risk Scores for Validating the Risk Score Model.

Supplementary Figures

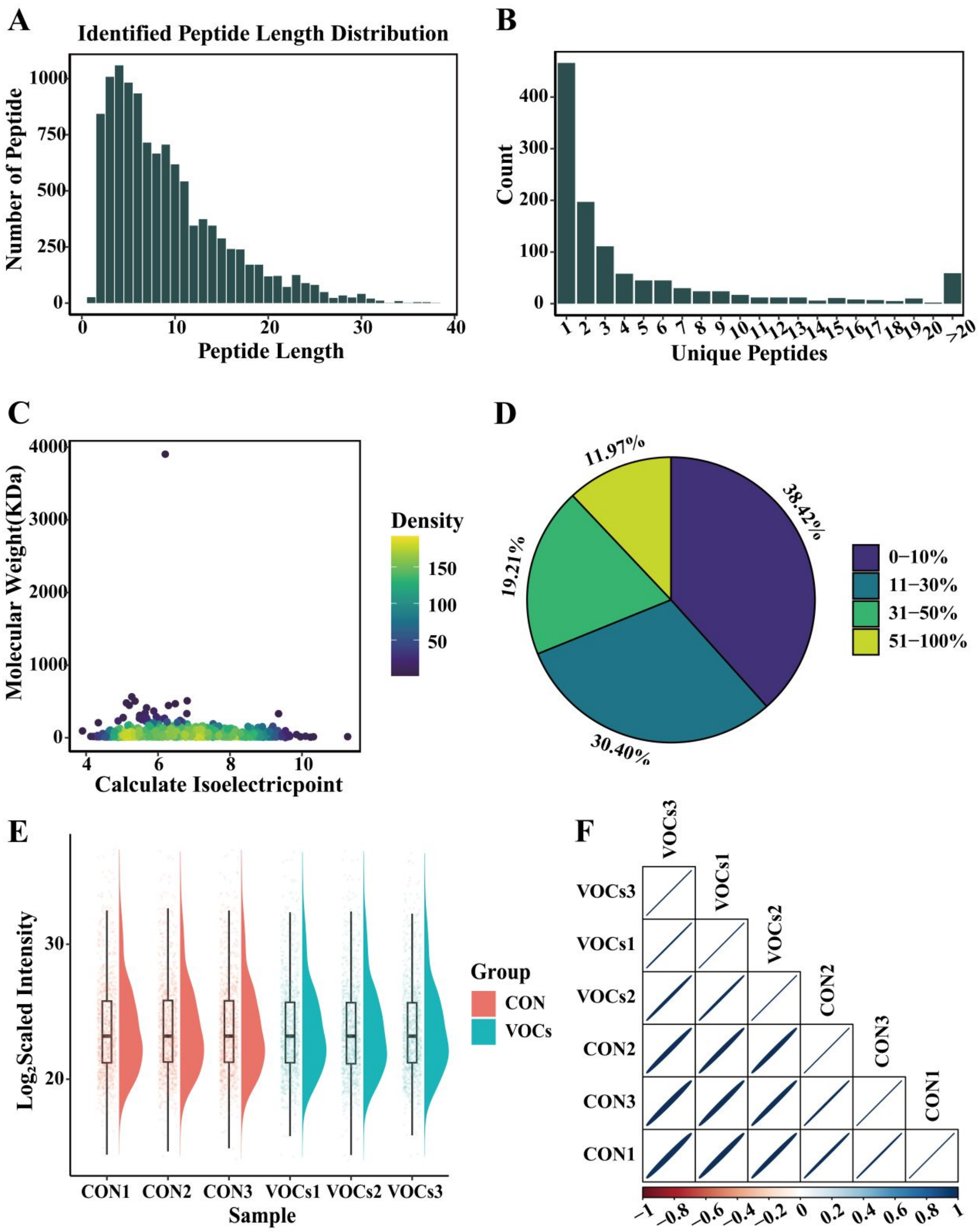

Figure S1. Data Quality Control in Proteomic Analysis. (A) Distribution of peptide length. (B) Distribution of the number of unique peptides. (C) Scatter plot of protein molecular weight versus isoelectric point. (D) Distribution of protein sequence coverage. (E) Distribution of protein expression abundance across samples. (F) Heatmap of Pearson correlation coefficients between samples.

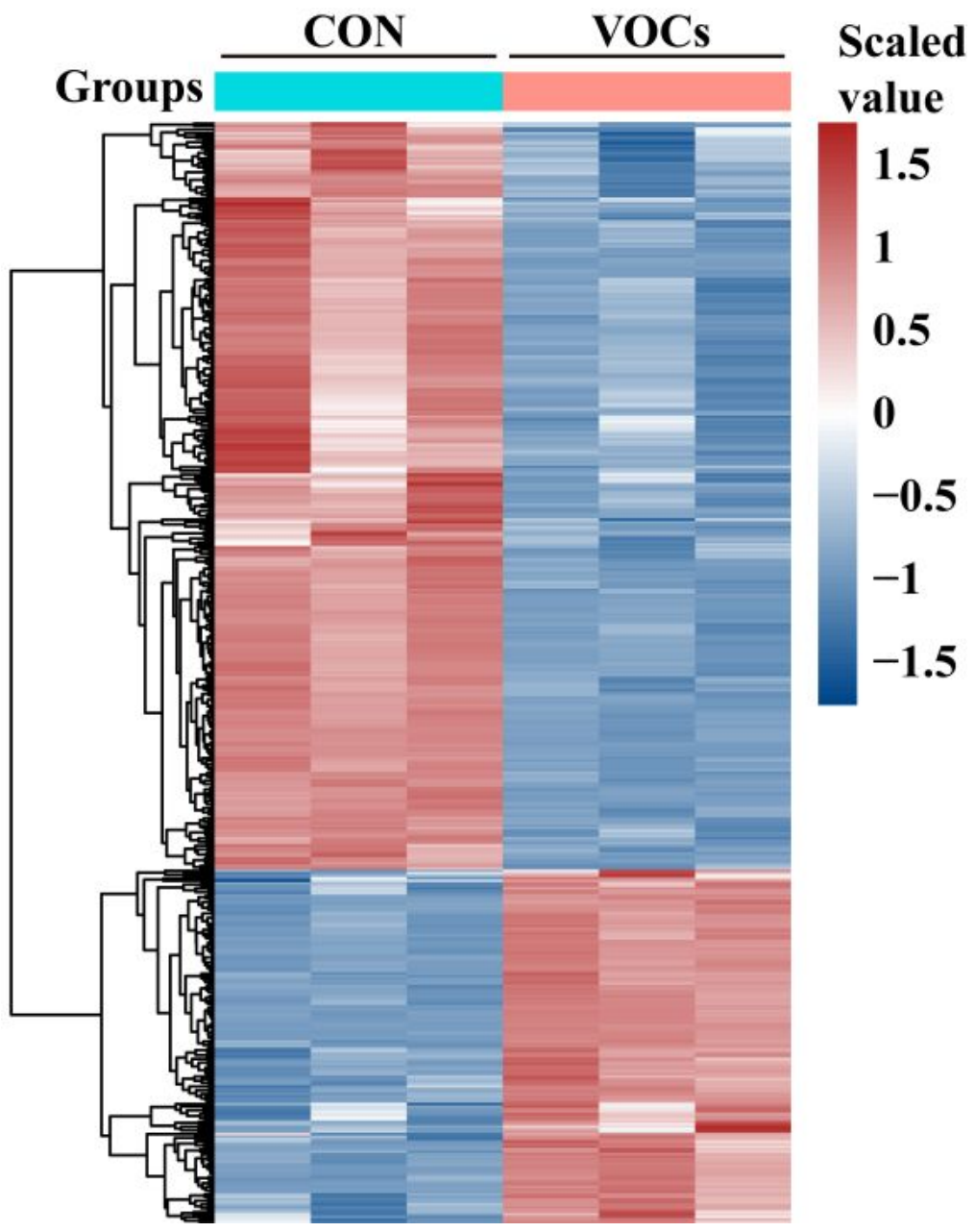

Figure S2. Heatmap of KEGG Pathway Enrichment Cluster Analysis for DEPs between the CON and VOCs Groups.

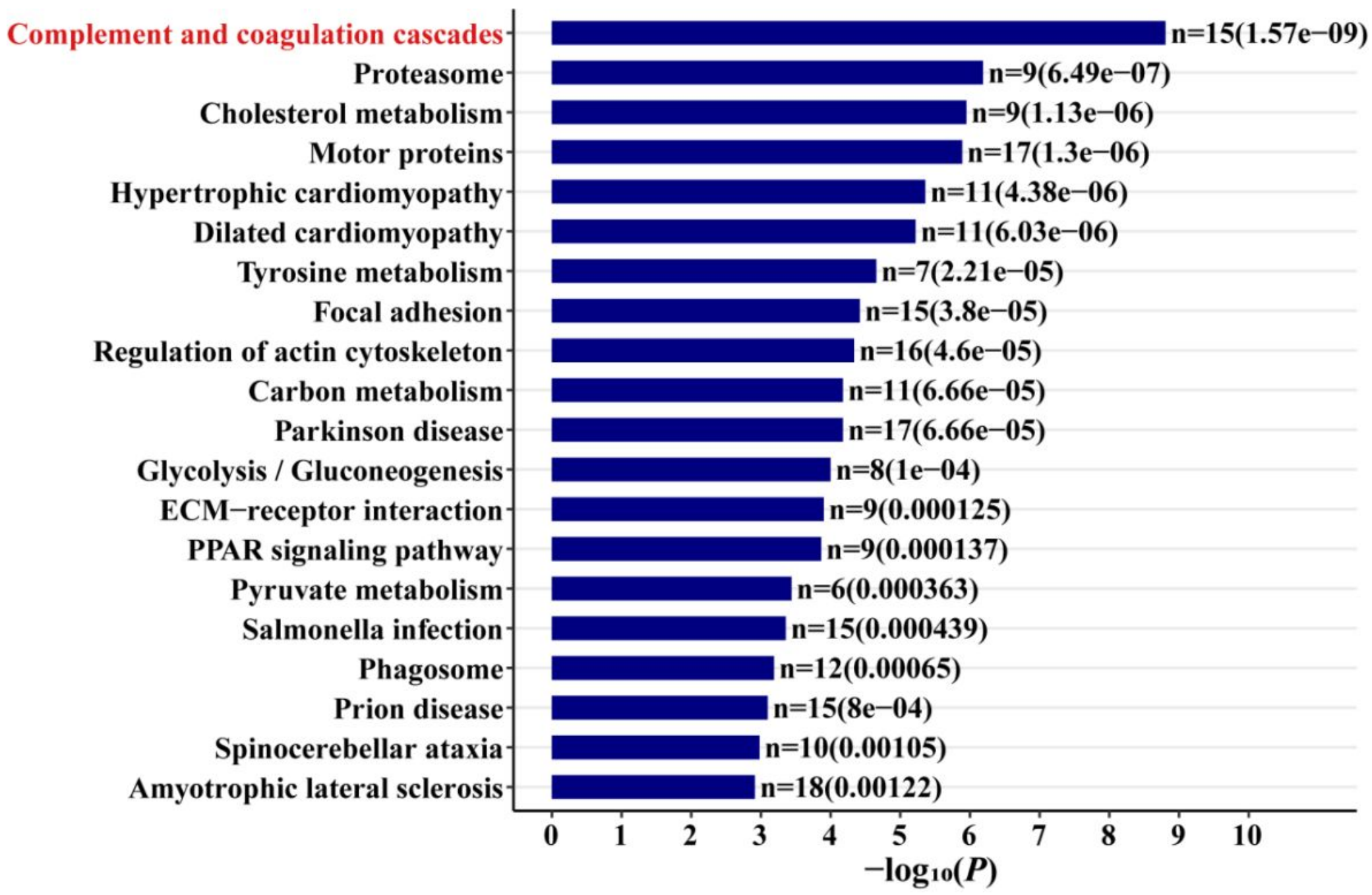

Figure S3. KEGG Pathway Enrichment Analysis of DEPs between the CON and VOCs Groups.

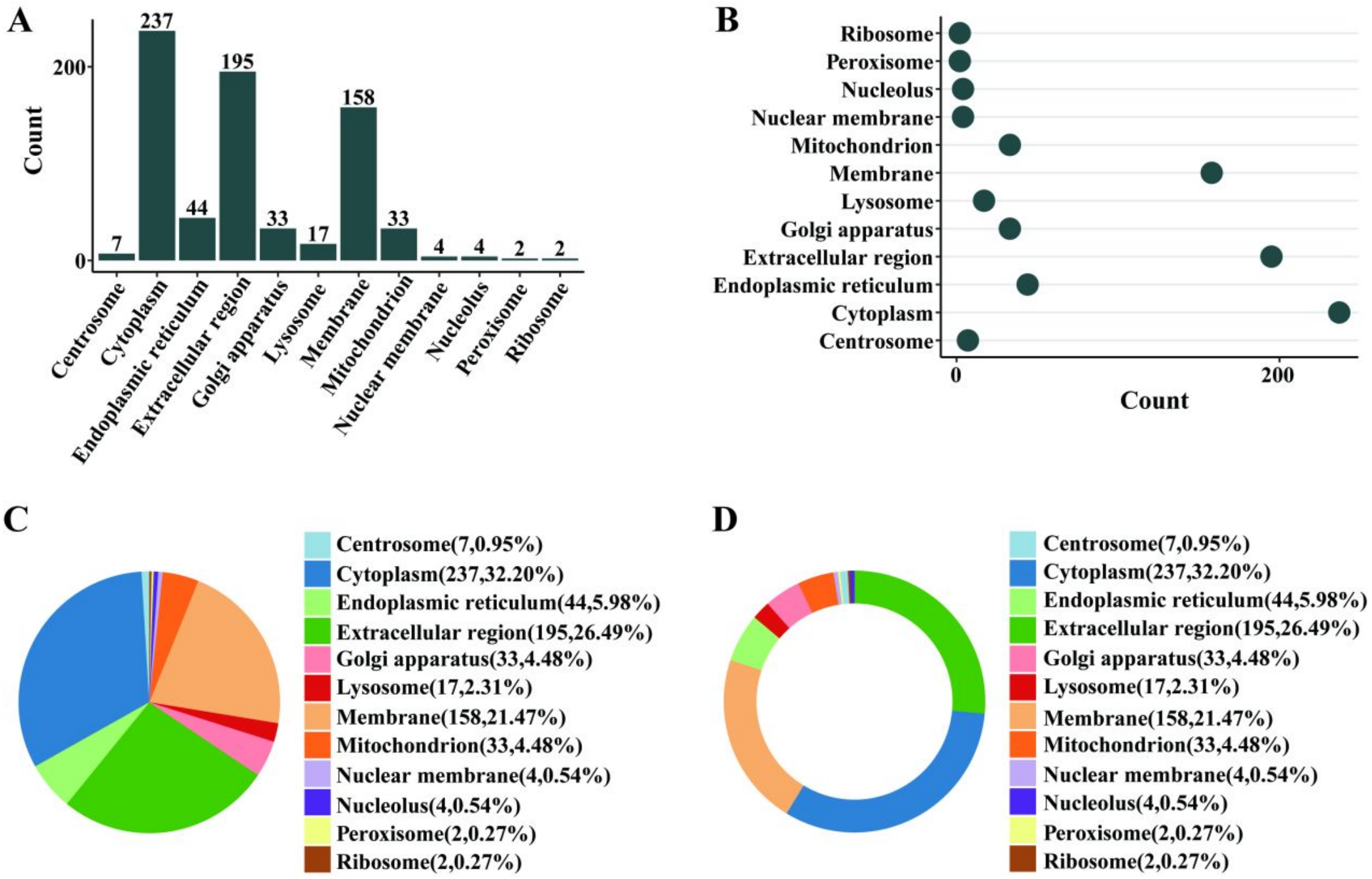

Figure S4. Subcellular Localization of DEPs between the CON and VOCs Groups. (A) Bar chart displaying the distribution of DEPs across subcellular compartments. (B) Bubble plot illustrating subcellular localization patterns of DEPs. (C) Pie chart showing the proportional distribution of DEPs in different subcellular locations. (D) Donut chart visualizing the percentage distribution of DEPs among various subcellular compartments.

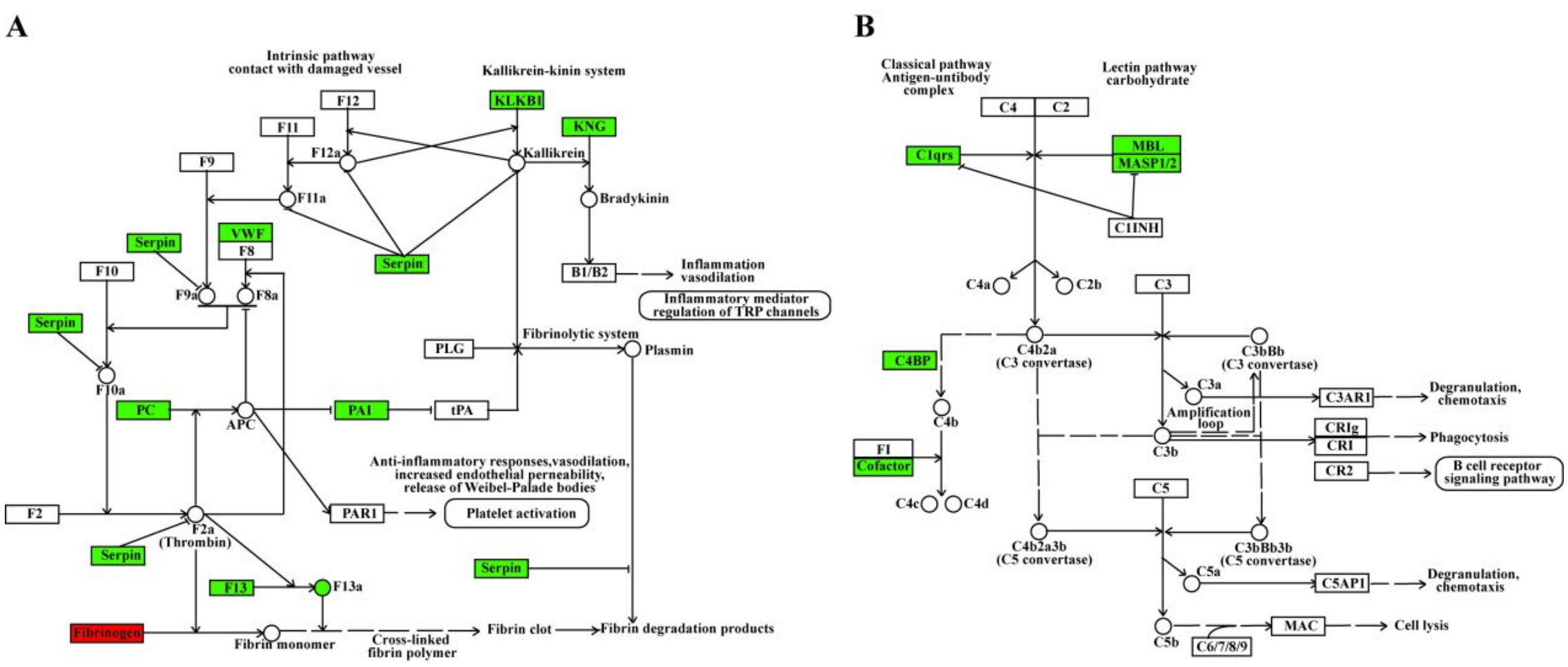

Figure S5. Potential Mechanisms Underlying Alterations in the Complement and Coagulation Cascades Following VOCs Exposure. (A) Schematic diagram of the coagulation system. (B) Schematic diagram of the complement system. Green boxes indicate downregulation, while red boxes represent upregulation.

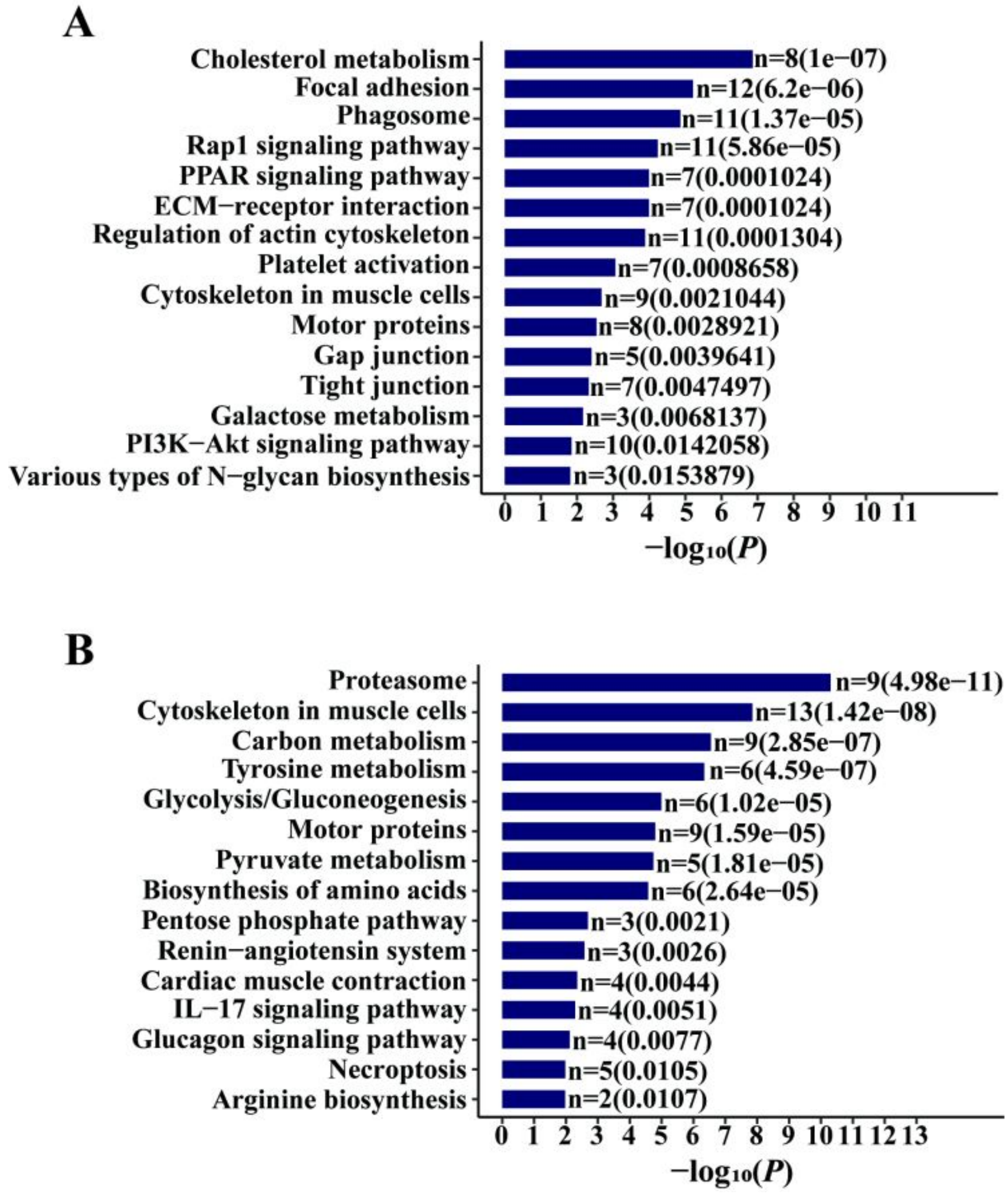

Figure S6. Enrichment Analysis of the Remaining Differentially Expressed Proteins After Excluding Those in the Complement and Coagulation Cascades. (A) KEGG functional enrichment bar plot for the remaining downregulated proteins, with pathways associated with human diseases also excluded. (B) KEGG functional enrichment bar plot for the remaining upregulated proteins, with pathways associated with human diseases also excluded. The x-axis represents $-\log_{10}(P)$.

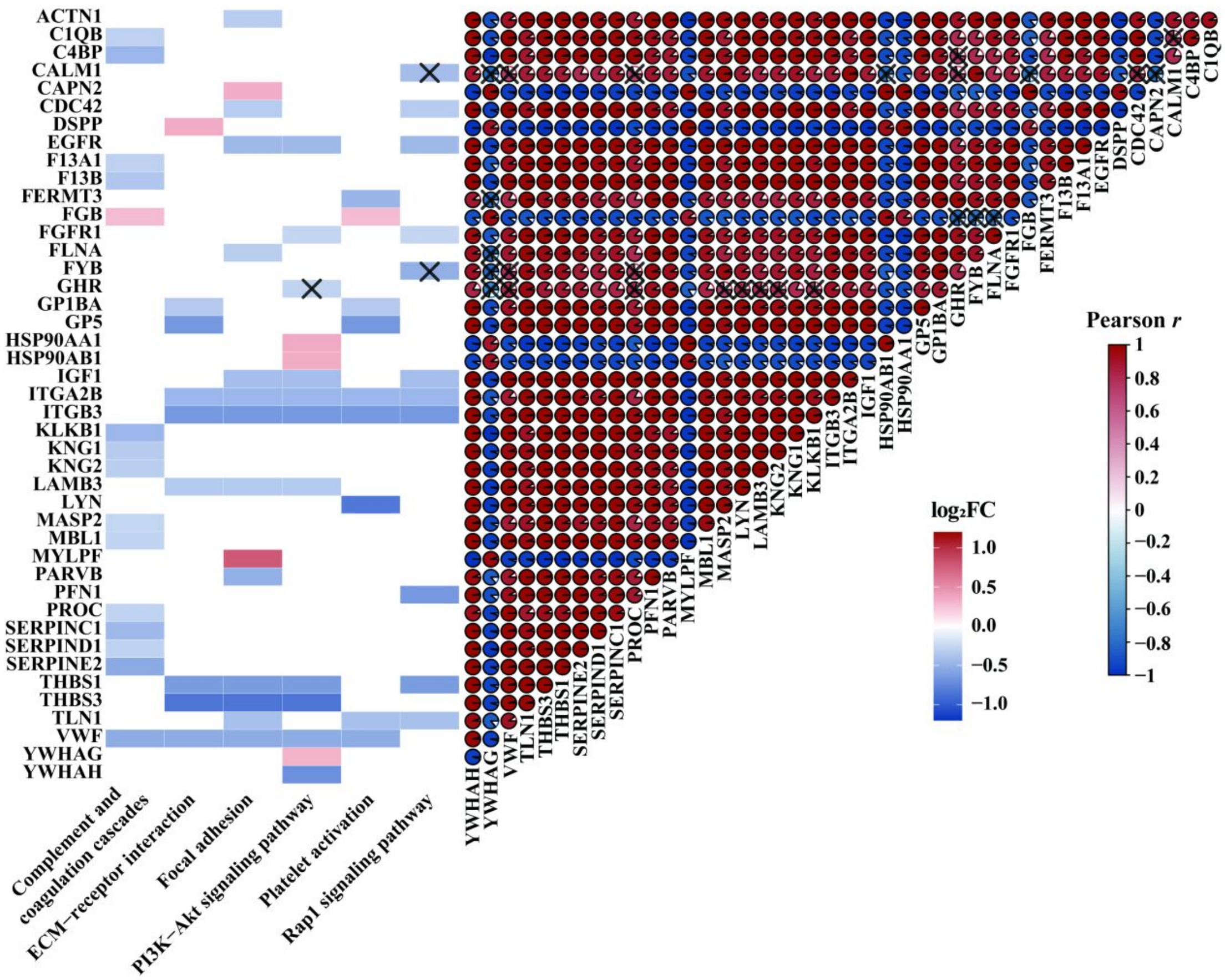

Figure S7. Correlation Analysis between DEPs in the Enriched Pathways of Downregulated and DEPs in the Complement and Coagulation Cascade. The cross symbol denotes the absence of statistical significance.

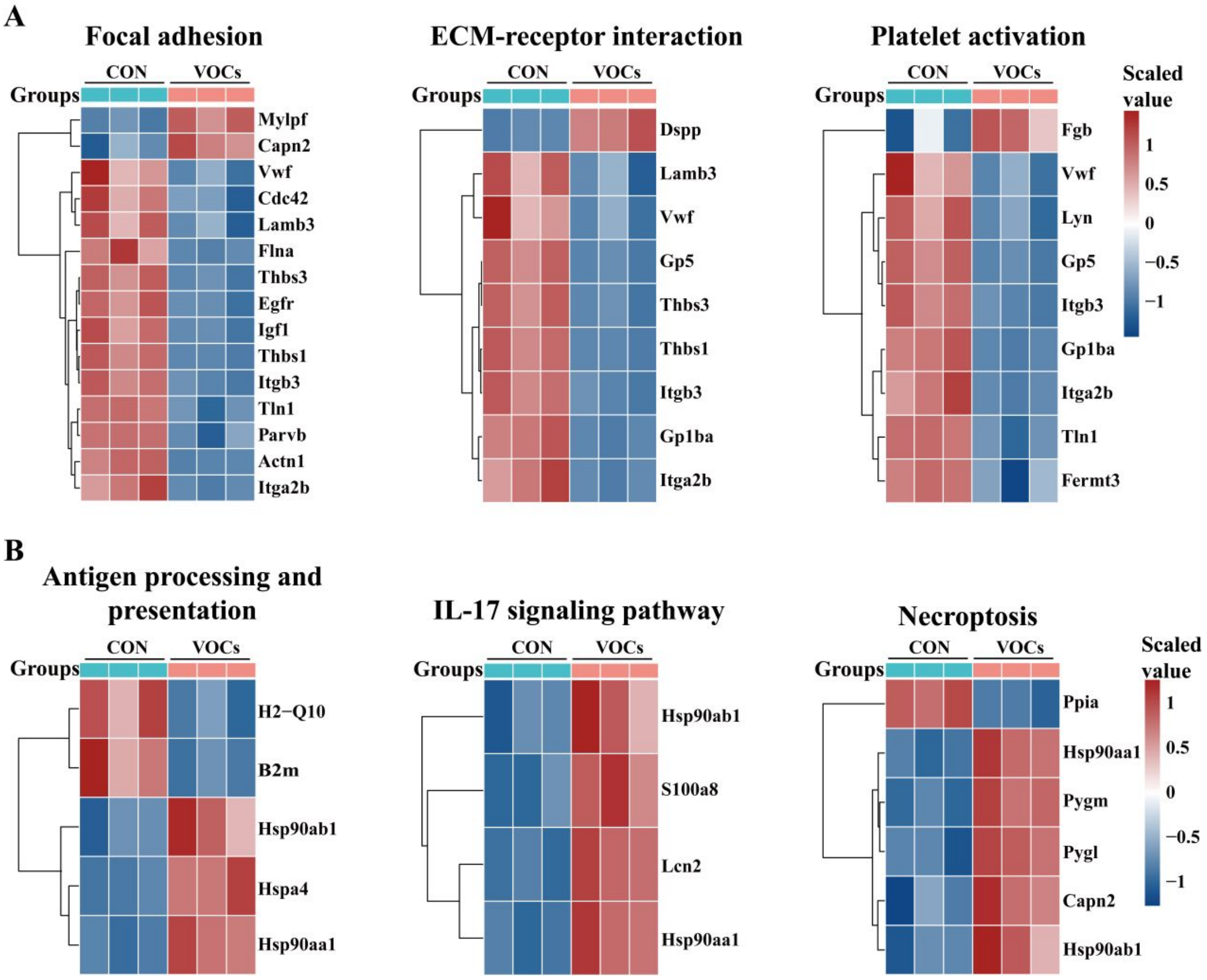

Figure S8. Alteration Profiles of Differentially Expressed Proteins in Key Pathways. (A) Heatmap of differentially expressed proteins enriched in key pathways among the remaining downregulated proteins. (B) Heatmap of differentially expressed proteins enriched in key pathways among the remaining upregulated proteins.

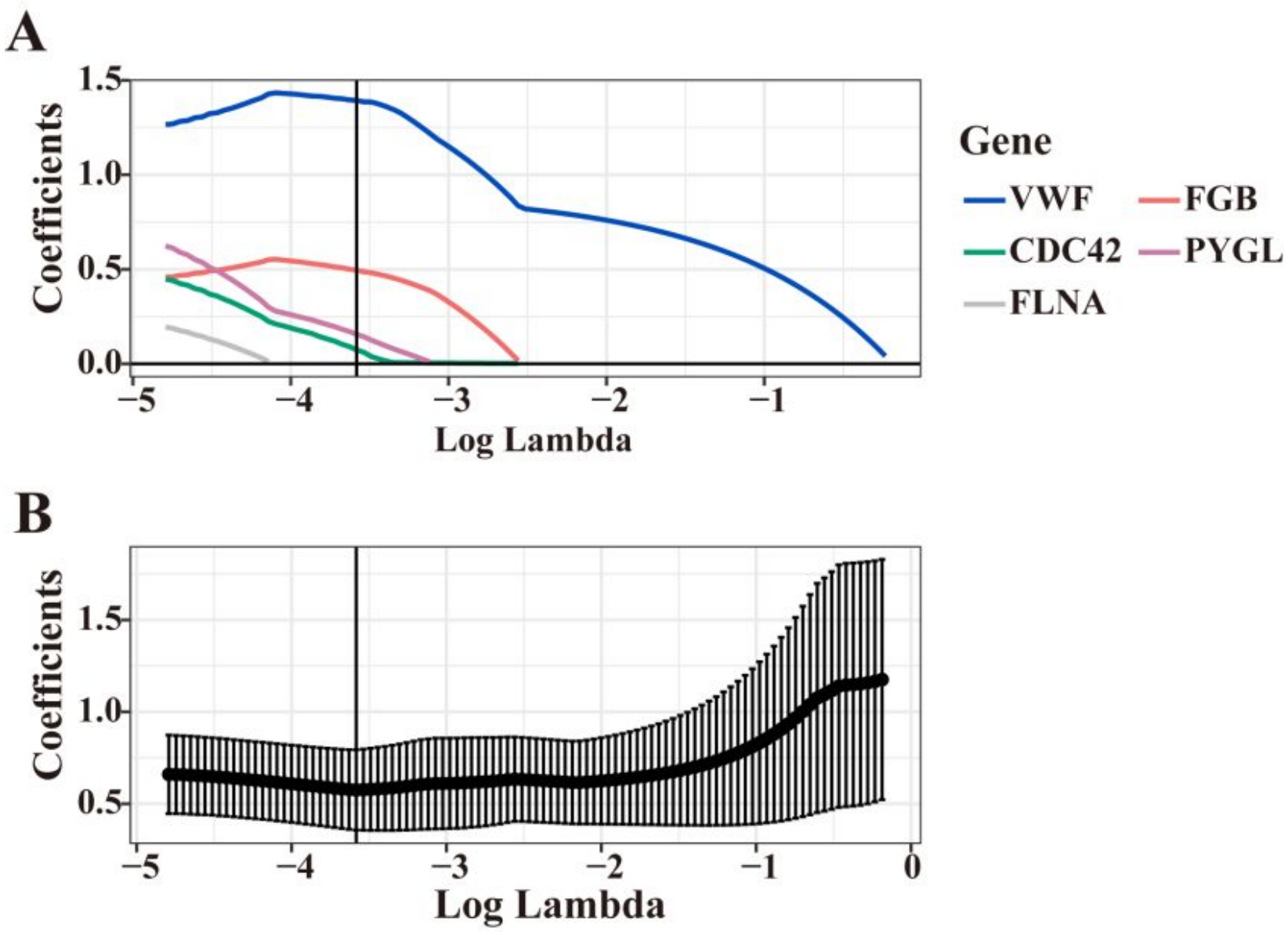

Figure S9. LASSO Analysis of Differentially Expressed Proteins in the Six Key Pathways. (A) Coefficient variation paths of four protein markers against the penalty parameter (λ). (B) Selection of the optimal tuning parameter (λ) in the LASSO model via ten-fold cross-validation using the minimum criterion.

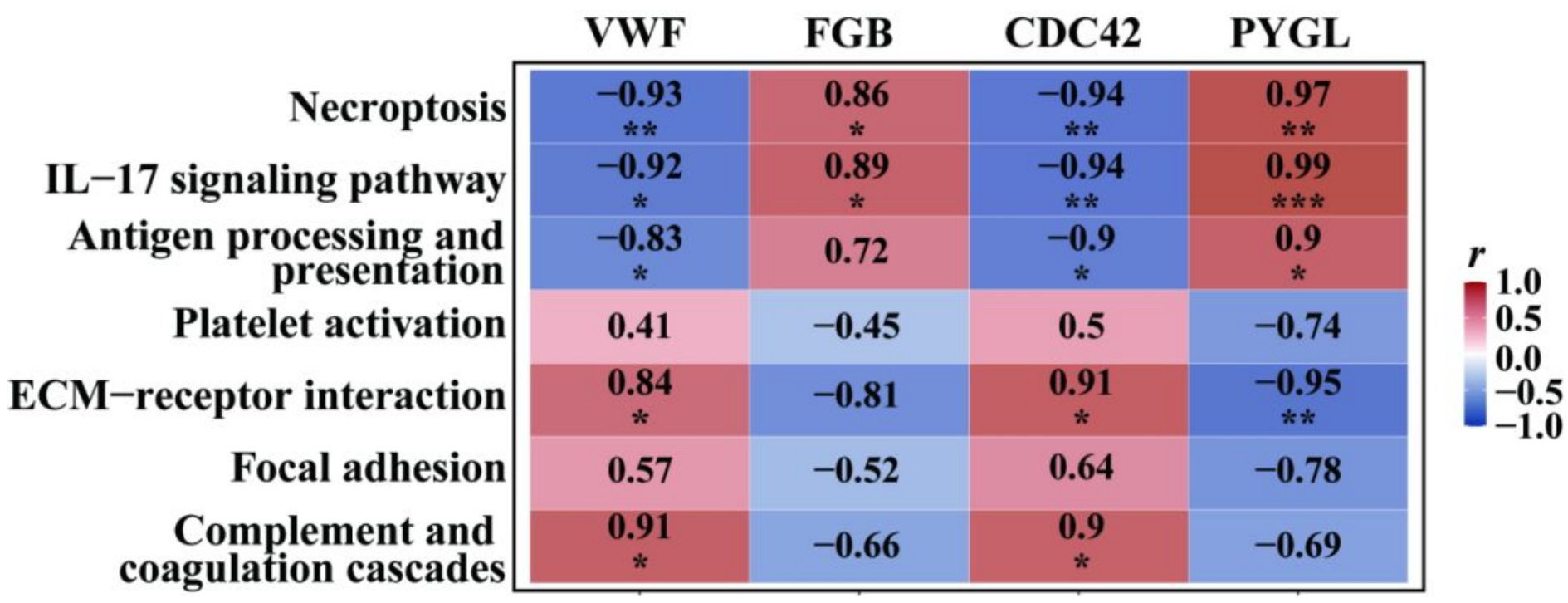

Figure S10. Correlation Analysis of Candidate Biomarkers with the Complement and Coagulation Cascades and Six Key Pathways. $^{*}P < 0.05$, $^{**}P < 0.01$, $^{***}P < 0.001$ (Pearson's correlation).

Supplementary Tables

Table S1. Reactome Pathway Analysis of DEPs between the CON and VOCs Groups.

The relationship with complement and coagulation	Pathway name	*P*
No significant correlation	Tyrosine catabolism	0.0002
No significant correlation	Phenylalanine and tyrosine metabolism	0.0021
No significant correlation	Defective visual phototransduction due to STRA6 loss of function	0.0022
Coagulation system	Intrinsic pathway of fibrin clot formation	0.0054
Complement system	Cross-presentation of soluble exogenous antigens (endosomes)	0.0102
Coagulation system and complement system	Neutrophil degranulation	0.0130
Weak correlation	VLDL clearance	0.0143
Complement system	Lectin pathway of complement activation	0.0143
Weak correlation	SCF-beta-TrCP mediated degradation of Emi1	0.0155
Weak correlation	Hh mutants are degraded by ERAD	0.0168

Table S2. GSEA of the Complement and Coagulation Cascades.

Description	Complement and coagulation cascades
EnrichmentScore	-0.3211
NES	-1.2906
P	0.0890

Table S3. Ranking of Degree Values for DEPs in Key Pathways and the Complement and Coagulation Cascades.

Rank	Name	Degree
1	VWF	19
2	ITGB3	18
3	THBS1	17
4	SERPINC1	16
5	ITGA2B	15
6	FGB	14
6	FLNA	14
6	PROC	14
9	SERPIND1	13
9	GP1BA	13
11	EGFR	12
11	KNG1	12
11	IGF1	12

Rank	Name	Degree
11	GP5	12
15	F13B	11
15	TLN1	11
17	HSP90AA1	10
17	KLKB1	10
17	SERPINE2	10
17	HSP90AB1	10
21	CDC42	9
21	C4BP	9
21	F13A1	9
21	MBL1	9
25	MASP2	8
25	LYN	8
25	B2M	8
28	PARVB	7
28	FERMT3	7
28	ACTN1	7
31	HSPA4	6
32	PPIA	4
32	C1QB	4
32	KNG2	4
35	THBS3	3
35	LCN2	3
37	CAPN2	2
37	MYLPF	2
37	S100A8	2
37	PYGM	2
37	LAMB3	2
42	H2-Q10	1
42	PYGL	1

Table S4. Correlation Analysis of VWF with the Complement and Coagulation Cascades and Key Pathways.

Category	Pathway	*r*	*P*
Complement /Coagulation	Complement and coagulation cascades	0.9081	0.0123
Suppression	Focal adhesion	0.5650	0.2427
	ECM-receptor interaction	0.8411	0.0359
	Platelet activation	0.4137	0.4149
Activation	Antigen processing and presentation	-0.8336	0.0392
	IL-17 signaling pathway	-0.9157	0.0104
	Necroptosis	-0.9302	0.0071

Table S5. LASSO Model Output Results.

LASSO	Variable	Coefficient (95% CI)
Model I	VWF	1.376 (0.779, 1.389)
	FGB	0.493 (0.000, 0.526)
	CDC42	0.092 (0.000, 0.188)
	PYGL	0.158 (0.000, 0.236)
Model II	VWF	0.870 (0.791, 0.873)
	CDC42	0.018 (0.000. 0.021)

Model I： Screening differential proteins in key pathways and complement and coagulation cascades.
Model II：Determining the weight coefficients of the final biomarkers.

Table S6. Risk Scores of the Risk Score Model.

Group	Risk score (95% CI)
CON	118350757 (90403864, 146297650)
VOCs	82054277 (69543037, 94565518)

Table S7. Protein Expression of VWF and CDC42.

Protein	Group	Concentration (± SD)
VWF (ng/μL)	CON	19241927 (± 2366673)
	VOCs	14265787 (± 1193818)
CDC42 (ng/μL)	CON	1059656 (± 76345)
	VOCs	773206 (± 56391)

Table S8. Risk Scores for Validating the Risk Score Model.

Group	Risk score (95% CI)
CON	16765422 (11645886, 21884958)
VOCs	12429501 (9849168, 15009833)
